# NMNAT2:HSP90 Complex Mediates Proteostasis in Proteinopathies

**DOI:** 10.1371/journal.pbio.1002472

**Published:** 2016-06-02

**Authors:** Yousuf O. Ali, Hunter M. Allen, Lei Yu, David Li-Kroeger, Dena Bakhshizadehmahmoudi, Asante Hatcher, Cristin McCabe, Jishu Xu, Nicole Bjorklund, Giulio Taglialatela, David A. Bennett, Philip L. De Jager, Joshua M. Shulman, Hugo J. Bellen, Hui-Chen Lu

**Affiliations:** 1 Linda and Jack Gill Center, Department of Psychological and Brain Sciences, Indiana University, Bloomington, Indiana, United States of America; 2 The Cain Foundation Laboratories, Texas Children’s Hospital, Houston, Texas, United States of America; 3 Jan and Dan Duncan Neurological Research Institute, Texas Children’s Hospital, Houston, Texas, United States of America; 4 Department of Pediatrics, Baylor College of Medicine, Houston, Texas, United States of America; 5 Rush Alzheimer’s Disease Center and Department of Neurological Sciences, Rush University, Chicago, Illinois, United States of America; 6 Department of Molecular and Human Genetics, Baylor College of Medicine, Houston, Texas, United States of America; 7 Department of Neuroscience, Baylor College of Medicine, Houston, Texas, United States of America; 8 Program in Medical and Population Genetics, Broad Institute, Cambridge, Massachusetts, United States of America; 9 Program in Translational NeuroPsychiatric Genomics, Institute for the Neurosciences, Departments of Neurology and Psychiatry, Division of Genetics, Department of Medicine, Brigham and Women’s Hospital, Boston, Massachusetts, United States of America; 10 Department of Neuroscience and Cell Biology, University of Texas Medical Branch, Galveston, Texas, United States of America; 11 Harvard Medical School, Boston, Massachusetts, United States of America; 12 Department of Neurology, Baylor College of Medicine, Houston, Texas, United States of America; 13 Program in Developmental Biology, Baylor College of Medicine, Houston, Texas, United States of America; 14 Howard Hughes Medical Institute (HHMI), Baylor College of Medicine, Houston, Texas, United States of America; Stanford University School of Medicine, UNITED STATES

## Abstract

Nicotinamide mononucleotide adenylyl transferase 2 (NMNAT2) is neuroprotective in numerous preclinical models of neurodegeneration. Here, we show that brain *nmnat2* mRNA levels correlate positively with global cognitive function and negatively with AD pathology. In AD brains, NMNAT2 mRNA and protein levels are reduced. NMNAT2 shifts its solubility and colocalizes with aggregated Tau in AD brains, similar to chaperones, which aid in the clearance or refolding of misfolded proteins. Investigating the mechanism of this observation, we discover a novel chaperone function of NMNAT2, independent from its enzymatic activity. NMNAT2 complexes with heat shock protein 90 (HSP90) to refold aggregated protein substrates. NMNAT2’s refoldase activity requires a unique C-terminal ATP site, activated in the presence of HSP90. Furthermore, deleting NMNAT2 function increases the vulnerability of cortical neurons to proteotoxic stress and excitotoxicity. Interestingly, NMNAT2 acts as a chaperone to reduce proteotoxic stress, while its enzymatic activity protects neurons from excitotoxicity. Taken together, our data indicate that NMNAT2 exerts its chaperone or enzymatic function in a context-dependent manner to maintain neuronal health.

## Abstract 2

This study reveals NMNAT2 to be a dual-function neuronal maintenance factor that not only generates NAD to protect neurons from excitotoxicity but also moonlights as a chaperone to combat protein toxicity.

## Author Summary

Pathological protein aggregates are found in many neurodegenerative diseases, and it has been hypothesized that these protein aggregates are toxic and cause neuronal death. Little is known about how neurons protect against pathological protein aggregates to maintain their health. Nicotinamide mononucleotide adenylyltransferase 2 (NMNAT2) is a newly identified neuronal maintenance factor. We found that in humans, levels of NMNAT2 transcript are positively correlated with cognitive function and are negatively correlated with pathological features of neurodegenerative disease like plaques and tangles. In this study, we demonstrate that NMNAT2 can act as a chaperone to reduce protein aggregates, and this function is independent from its known function in the enzymatic synthesis of nicotinamide adenine dinucleotide (NAD). We find that NMNAT2 interacts with heat shock protein 90 (HSP90) to refold protein aggregates, and that deleting NMNAT2 in cortical neurons renders them vulnerable to protein stress or excitotoxicity. Interestingly, the chaperone function of NMNAT2 protects neurons from protein toxicity, while its enzymatic function is required to defend against excitotoxicity. Our work here suggests that NMNAT2 uses either its chaperone or enzymatic function to combat neuronal insults in a context-dependent manner. In Alzheimer disease brains, NMNAT2 levels are less than 50% of control levels, and we propose that enhancing NMNAT2 function may provide an effective therapeutic intervention to reserve cognitive function.

## Introduction

Robust neuronal maintenance mechanisms are required to minimize or repair damage arising from intrinsic and extrinsic stressors. Nicotinamide mononucleotide adenylyl transferases (NMNATs) play important roles in neuronal maintenance in flies [1,2] and human [3–6], and their overexpression provides neuroprotection in diverse neurodegenerative models [7,8]. NMNATs are known as nicotinamide adenine dinucleotide (NAD)-synthesizing enzymes that enable proper flux of NAD, an essential cofactor for many cellular processes [7,8]. The literature regarding the importance of this enzymatic activity in axonal health is mixed [8]. The majority of evidence that supports the importance of NMNAT’s enzymatic activity comes from studies using Wallerian degeneration models. Upon nerve cut, NAD levels are quickly reduced, and exogenous NAD application offers protection [9–12]. Abolishing NMNAT enzymatic function impairs their axon-protective function against injuries [9,10,12–14]. However, NMNAT enzymatic activity is not required to maintain neural integrity in *Drosophila* photoreceptors [2], suggesting that NMNATs may protect neurons by different mechanisms in a context-dependent manner [7,8].

NMNAT2 has the shortest half-life among three mammalian NMNATs and its function in axonal survival cannot be compensated by endogenous NMNAT1 or 3 [15,16]. The constant axonal supply of NMNAT2 from Golgi-derived vesicles is critical to maintain axonal health [16,17]. Constitutive NMNAT2 removal results in neurite outgrowth deficits [18,19], while knocking down NMNAT2 in developed neurons causes axonal degeneration [15], a prominent feature of many neurodegenerative diseases [20–23]. Importantly, NMNAT2 is highly expressed in the mammalian brain [24,25]. *nmnat2* mRNA levels are reduced in Parkinson, Huntington, and Alzheimer diseases (AD), as well as in tauopathies [26–31], suggesting a role of NMNAT2 in maintaining neuronal health in the aging brain. In rTg4510 transgenic mice, a Frontotemporal Dementia and Parkinsonism-17 (FTDP-17) tauopathy model, NMNAT2 abundance declines prior to the onset of neurodegeneration or memory deficits [32]. Moreover, elevating NMNAT2 levels in rTg4510 mice ameliorates their neurodegenerative phenotype. These finding suggest a role for NMNAT2 in neuronal maintenance in the brain, but the mechanism underlying neuroprotection remains to be elucidated.

*Drosophila* NMNAT (*d*NMNAT) also functions as a molecular chaperone [2]. Molecular chaperones are defined as a class of proteins that interact with, stabilize, or assist proteins to retain their native and functionally active conformation [33]. They are critical in maintaining protein homeostasis (proteostasis) and to facilitate the clearance of pathological protein aggregates [34,35]. Furthermore, the identification of synaptic chaperones has highlighted the importance of chaperones in preserving neuronal function [36–40]. For example, the synaptic vesicle protein cysteine string protein α (CSPα) forms a chaperone complex with heat shock cognate 70 (Hsc70) and the small glutamine-rich tetratricopeptide repeat protein SGT to promote SNARE-complex assembly and maintain the presynaptic release machinery [37,38]. Haploinsufficency of CSPα in humans causes autosomal dominant adult onset neuronal ceroid lipofusinosis and leads to dementia [41,42].

Proteinopathies, including AD and tauopathies, are characterized by stereotypic aggregated proteins, and this pathology is associated with cognitive impairment [43,44]. It has been proposed that enhancing chaperone activity helps to establish a cytoprotective state, defending against the cellular damage caused by misfolding and/or aggregation associated proteinopathies [45]. The role of chaperones in AD has been studied extensively with respect to tau aggregation and fibrillization. Chaperones, such as HSP70, HSP90, and the C-terminus of HSC70 Interacting Protein (CHIP) have been shown to preferentially bind to hyperphosphorylated human Tau (p-hTau) as well as paired helical filamentous tau, but not to nonphosphorylated tau [46]. Indeed, overexpression of cytosolic HSP70 and HSP90 inhibit the early stages of amyloid aggregation in AD models [47]. The reduced Tau burden upon elevating NMNAT2 levels in rTg4510 mice [32] prompted us to ask the following three questions. Does NMNAT2 act as a chaperone? Is this chaperone activity required for NMNAT2 to reduce tauopathy and protect neurons against protein stress? How does NMNAT2 exert its chaperone function?

In this study, we validate the clinical relevance of human NMNAT2 levels by examining the relationship between *nmnat2* mRNA levels and the cognitive capabilities and AD pathology in a large cohort of aged human subjects. In AD brains, NMNAT2 shifts into the insoluble fraction together with chaperones like HSP70 [48–51]. Next, we show that NMNAT2 functions as a chaperone in several in vitro and in vivo assays. We define the domain that acts as a chaperone and show that NMNAT2 acts with HSP90 to clear protein aggregates. Furthermore, we demonstrate that NMNAT2’s enzymatic or chaperone function is differentially recruited to protect neurons depending on the nature of insult. Deleting NMNAT2 in cortical neurons increases their vulnerability to proteotoxic stress and excitotoxicity triggered by excessive neurotransmission. The chaperone function of NMNAT2 is required to defend against proteotoxic stress, while its enzymatic function is indispensible for maintaining viability under excitotoxic conditions. Taken together, our findings suggest that the dual-functions of NMNAT2 protect neurons and preserve cognitive function during aging.

## Results

### NMNAT2 mRNA Levels Positively Correlate with Global Cognitive Function and Negatively with AD Pathology

**Fig 1:**
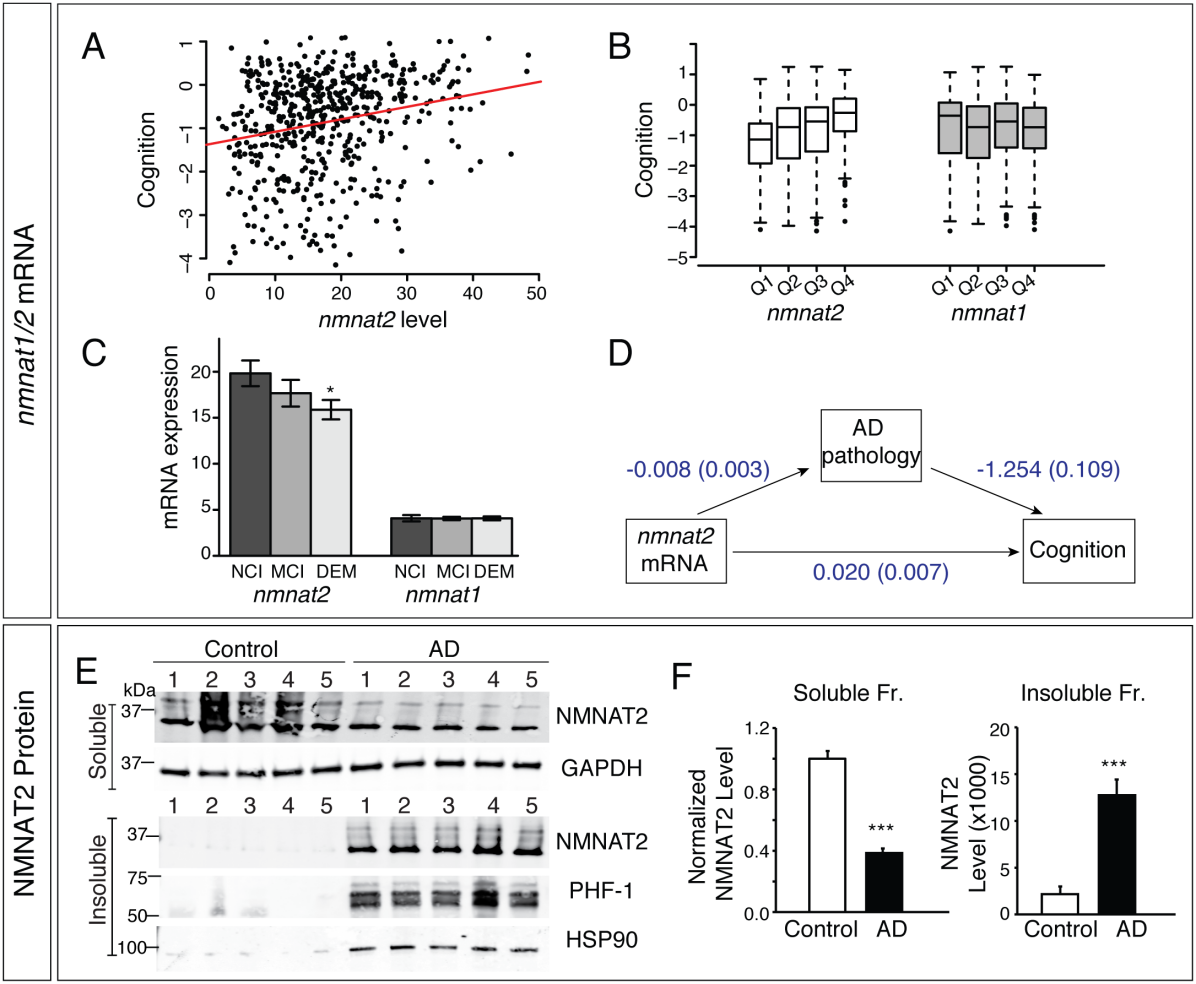
NMNAT2 expression in human brain positively correlates with global cognition scores. (**A**) The scatter plot shows individual subject values for *nmnat2* mRNA levels and global cognition scores proximate to death. The regression line shows the positive relationship between *nmnat2* levels and cognitive scores. Units for both mRNA and cognitive scores are arbitrary (Materials and Methods for details). (**B**) Box plots show global cognition scores within each quartile of *nmnat1/2* level. Each box is defined by the interquartile range, the line in the box is the median, and the whiskers are 1.5*interquartile range. (**C**) Bar graphs of *nmnat2* and *nmnat1* mRNA levels by clinical diagnosis. Abbreviations: NCI, no cognitive impairment; MCI, mild cognitive impairment; DEM, dementia. (**D**) Path analysis of hypothetical structural models linking *nmnat2* levels with cognition, either indirectly via an effect on AD pathology (top) or directly (bottom). The arrows in the model represent the hypothetical causal directions of the effects being tested by the statistical modeling. Standardized path coefficient (standard error) is shown, revealing that 30% of the NMNAT2 effect on cognition is mediated by AD pathologic burden. (**E–F**) NMNAT2 protein levels were reduced in the soluble fractions of AD brains. The insoluble fraction contains aggregated proteins such as insoluble Tau (revealed by PHF-1 antibody, which detects p-S396/404 hTau). NMNAT2 and HSP90 shift solubility in AD brains, appearing in the insoluble fraction. *,*** indicate *p* < 0.05, *p* < 0.001. The authors confirm that all data underlying the findings are fully available without restriction. Data for Fig 1E and 1F can be found in S1 Data. Data for Fig 1A–1C are available upon request via an online request tool listed in http://www.rush.edu/radc.

To evaluate the clinical relevance of NMNAT2 in human brain, we examined postmortem brain tissue and data available from the Religious Orders Study and the Rush Memory and Aging Project [52–54]. These studies combine longitudinal clinical and cognitive evaluations with brain neuropathological evaluations at death. A global cognitive summary measure is based on subject performance on a battery of 17 standardized cognitive tests, while a global AD neuropathologic burden is computed from counts of neuritic plaques and neurofibrillary tangles on silver-stained tissue sections from five brain regions [55,56]. *nmnat1/2* mRNA levels were extracted from an RNA-sequencing dataset profiling the transcriptome in dorsolateral prefrontal cortex samples from 541 deceased subjects (mean age at death = 88.4 y, standard deviation (sd) = 6.7; S1 Table). Higher *nmnat2* mRNA levels are associated with better cognitive performance proximate to death (*p* = 0.0007; Fig 1A–1D) as well as with a lower AD neuropathological burden (*p* = 0.004; S2 Table). These associations are robust when adjusted for age of death, postmortem interval and RNA quality (RNA integrity number; RIN) (S1 Table). By contrast, there is no relationship between *nmnat1* mRNA levels with cognition or AD pathology in this cohort (Fig 1B and 1C, S2 Table).

Causality cannot be inferred from such human data analysis. Path analysis, developed by the geneticist Sewall Wright [57], is a standard approach in human genetics to examine the complex interrelationships among multiple variables and to assess the relative importance of these variables upon outcome(s) of interest, including a range of potential mediators [57–60]. We conducted path analysis to examine the interrelationship between *nmnat2* expression, AD pathology and cognition. Fig 1D illustrates our hypothetical structural model, including a direct path that links *nmnat2* expression to cognition, as well as an indirect path that links its expression to AD pathology and, subsequently, to cognition. Standardized path coefficients and the corresponding standard error for each path were reported to assess both the hypothesized direct and indirect effect of *nmnat2* expression on cognition, using standard notation [60]. The relations represented by arrows in Fig 1D reflect our a priori hypothesis and are consistent with, but are not proof of, causality. We find that about 30% of the effect of *nmnat2* mRNA abundance on cognition is explained by global AD pathology (*p* = 0.003). This result is consistent with a hypothetical model in which at least part of the relation between *nmnat2* and cognition is mediated by AD pathology. The placement of AD pathology downstream of *nmnat2* expression in this model suggests the role of NMNAT2 in modulating AD pathology.

An independent cohort of human cortical samples consisting of control and AD brains were acquired from the Oregon Brain Bank. In this cohort, we observed a significant reduction of *nmnat2* mRNA as well as NMNAT2 protein but not NMNAT1 (S1 Fig; S3 Table). Soluble and insoluble proteins were extracted sequentially from human postmortem brain tissues with progressively more stringent detergents thereby fractionating human brain proteins based on their solubility [61,62], allowing the more soluble proteins to be extracted first. This was followed by the extraction of membrane-bound proteins, and finally the most insoluble proteins were isolated in a 2% SDS buffer. Aggregated proteins such as neurofibrillary tangles and amyloid plaques are typically present in insoluble fractions prepared from AD brains [63]. The majority of NMNAT2 in control brains was extracted in the soluble fraction (Fig 1E and 1F), while very little NMNAT2 was found in the insoluble fraction. However, in brains of AD patients, abundant NMNAT2 protein was detected in the insoluble fraction (*p* < 0.001). This fraction also contains hyperphosphorylated hTau and HSP90 (Fig 1E). The strong shift in solubility of NMNAT2 in AD brains is reminiscent of the behavior of chaperones, such as HSP70, HSP90 and HSP27, and cochaperones such as CHIP, which have been linked to pathological aggregates in AD [64].

### NMNAT2 Functions as a Chaperone with Both Holdase and Foldase Activities

**Fig 2:**
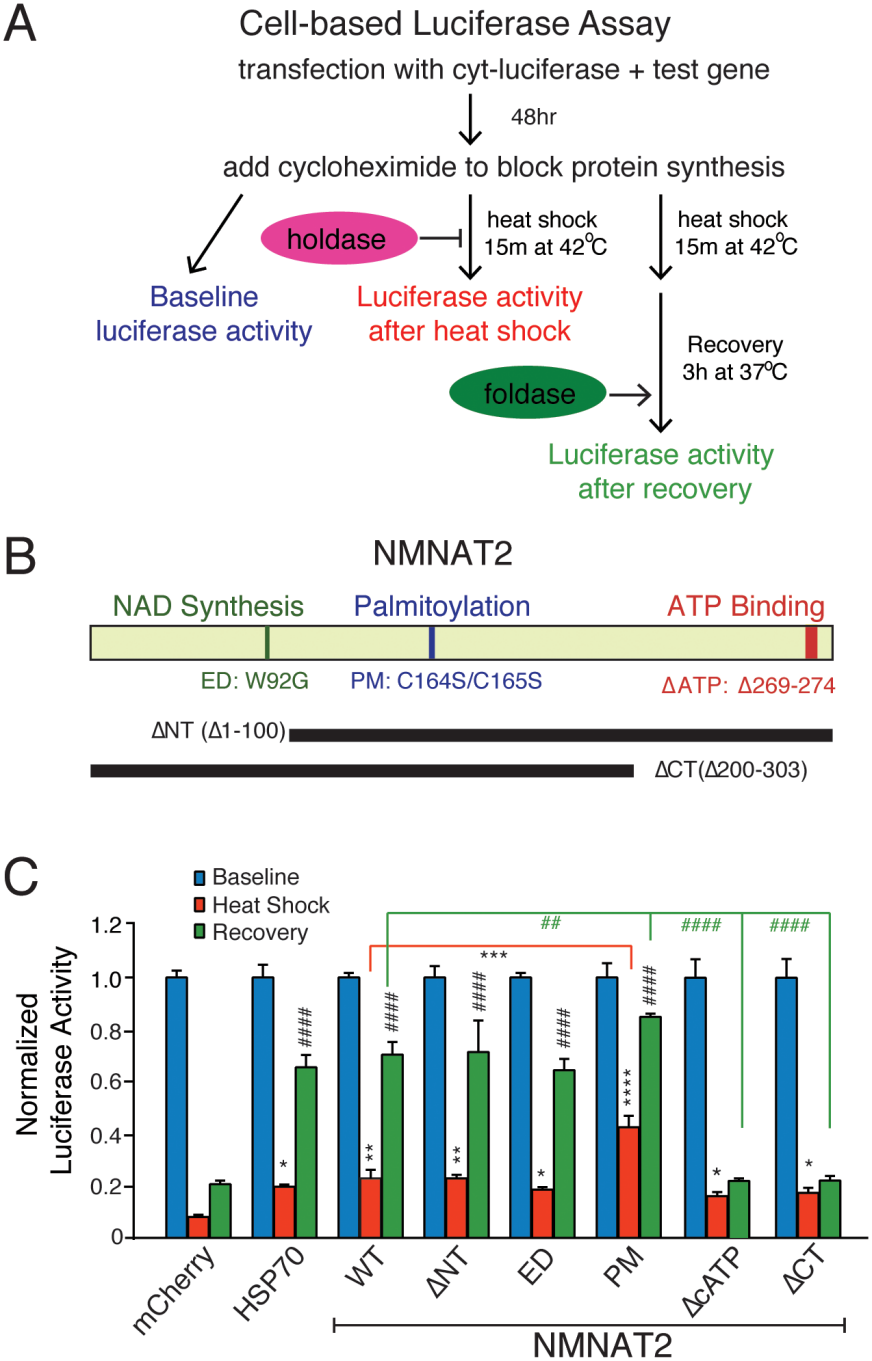
NMNAT2 exerts chaperone activity independently from its NAD-synthase function. **(A)** Diagram illustrates simplified experimental procedure of cell-based luciferase denaturation and refolding assay. **(B)** Diagram showing human NMNAT2 and the mutants generated for this study. **(C)** Summary for the chaperone activity of mCherry, HSP70, and various NMNAT2 mutants. Blue bars show baseline luciferase activity. Red bars show luciferase activity immediately after heat shock, while blue bars show luciferase activity after recovery. * and # indicate significant differences from mCherry heat shock, and mCherry recovery, respectively (*n* = 3 with triplicates per experiment). Individual values for 2C are provided in S1 Data. *^/#^,**^/##^,***^/###^,****^/####^ indicate *p* < 0.05, *p* < 0.01, or *p* < 0.001, *p* < 0.0001, respectively.

To assess the chaperone activity of human NMNAT2, we first conducted a cell-based luciferase assay [65,66]. In this assay, treatment with cycloheximide (200 uM for 3 h) to block protein synthesis, followed by heat denaturation (at 42°C for 15 min), renders the endogenous chaperone machinery incapable of preventing luciferase aggregation and refolding (Fig 2A). This allows for a direct measurement of the chaperone activity of the introduced test protein by its ability to prevent luciferase from undergoing heat shock-induced denaturation (holdase activity) and to promote proper refolding (foldase activity) of heat-denatured luciferase during recovery (at 37°C for 3 h). We found that NMNAT2’s holdase and foldase activity are comparable to that of HSP70 (S2 Fig). In other words, NMNAT2 reduces luciferase aggregation upon thermal stress and promotes its refolding, as indicated by higher luciferase activity postrecovery in NMNAT2 overexpressing cells.

To assess if NMNAT2 can directly bind to substrates and reduce protein aggregation in the absence of other proteins, a cell-free chaperone activity assay was performed. In the presence of thermal stress, citrate synthase (CS) aggregates leading to increased optical absorbance that can be detected as a result of Raleigh scattering [67]. This assay with recombinant proteins allows us to examine whether NMNAT2 can exert holdase activity to maintain CS in a thermostable conformation in the absence of other chaperones, cochaperones, or ATP. Both NMNAT2 and HSP70, but not lysozyme, are capable of preventing thermally-induced CS aggregation in a dose-dependent manner (S3 Fig).

### The Enzymatic and Chaperone Function of NMNAT2 Are Mediated by Different Protein Domains

Homology-based structural modeling of NMNAT2 shows that both the N- and C-terminal domains are highly conserved among NMNATs [24], while the central domain of NMNAT2 is different [68]. Both NAD synthesis activity [68,69] and chaperone function are comparable among NMNAT1-3 (S2 and S3 Figs). Previous studies have shown that mutating W92G eliminates more than 95% of NMNAT2’s catalytic activity [14,70] whereas mutating two palmitoylation sites (Cys-164 and Cys-165) in the central region of the protein increases stability [16,17]. We therefore generated an enzyme-dead (ED) NMNAT2 by mutating residue W92 (NMNAT2_W92G_; ED) or by deleting residues 1 to 100 of the N-terminus (Δ1–100; ΔNT) to entirely eliminate NMNAT2’s NAD catalytic activity (Fig 2B, S4A and S4B Fig). As shown in Fig 2C, both NMNAT2-ΔNT (*p* = 0.0004) and -ED (*p* = 0.0098) retained the ability to decrease luciferase aggregation and increase refolding similarly to NMNAT2-WT and HSP70.

Mutating the palmitoylation sites at Cys-164 /Cys-165 to serines (C164S;C165S; PM) significantly improved enzymatic (*p* < 0.001) and chaperone activity (*p* < 0.0001) for either holdase or foldase activity (Fig 2C) in addition to its stability (S4C Fig). However, deleting the C-terminal domain (Δ200–308; ΔCT) severely impaired NMNAT2’s foldase (*p* < 0.0001) but not its holdase activity (*p* = 0.87, compared to WT; Fig 2C), suggesting that this domain is selectively required for foldase activity. ATP binding has been shown to enhance chaperone activity of the large heat shock protein families by suppressing protein unfolding and aggregation [35,71–74]. The C-terminal region of NMNAT is predicted to contain an ATP-binding site spanning residues 269–274. These residues are highly conserved among NMNATs. Deleting 5 aa in the C-terminal ATP site (Δ269–274; ΔcATP) renders NMNAT2 incapable of promoting luciferase refolding (*p* < 0.0001, Fig 2C) but does not affect NAD synthesis (*p* = 0.79, S4A and S4B Fig). The deficits in foldase activity observed in NMNAT2-ΔCT and -ΔcATP are unlikely to be caused by alterations in their stability, as the half-lives of these proteins are similar to NMNAT2-WT, -ΔNT, and -ED (S4C Fig). Hence, our data indicate that the C-terminal ATP site is critical for NMNAT2’s foldase function, and the domains implicated in NAD enzymatic and chaperone function are mutually exclusive.

### The Chaperone Function of NMNAT2 Is Required to Reduce pTau

**Fig 3:**
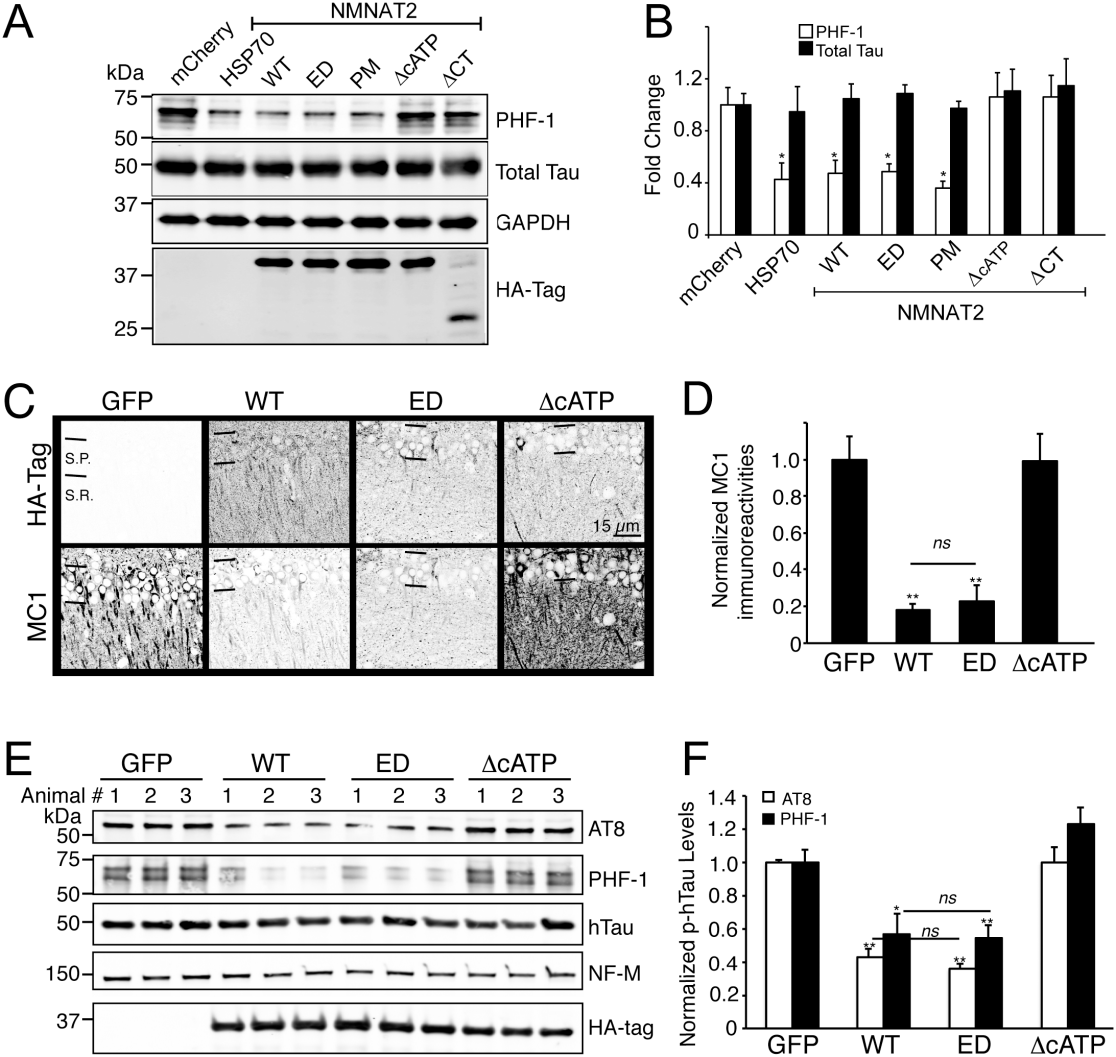
NMNAT2’s chaperone activity is required to reduce p-hTau levels both in vitro and in vivo. (**A–B**) Example western blot shows an increase of p-hTau in an inducible hTau40 cell line after doxycycline treatment. The increase in p-hTau was prevented by HSP70, NMNAT2-WT, -ED, or -PM but not -ΔCT or -ΔcATP (*n* = 4 independent experiments). (**C**) Overexpression of NMNAT2-WT or -ED but not -ΔcATP, reduced the levels of p-hTau revealed by MC1 immunoreactivity in the CA1 region of the hippocampus (GFP, *n* = 8; WT, *n* = 6; ED, *n* = 5; ΔcATP, *n* = 6). (**D**) Summary of normalized MC1 immunoreactivity in the CA1 S.P. area. (**E**) Representative western blots show the levels of p-hTau species recognized by PHF-1 or AT8 antibodies, neurofilaments (NF-M), total hTau, and HA-tag (recognizes exogenous NMNAT2). (**F**) p-hTau levels were quantified and normalized to sample neurofilament levels. Individual values for 3B, 3D and 3E are provided in S1 Data. S.P, striatum pyramidale layer; S. R., striatum radiatum layer; ns, not significant.

Intracellular aggregation of the microtubule-associated protein tau into filamentous inclusions is a defining characteristic of AD. Because the appearance of tau-aggregate bearing lesions correlates with both cognitive decline and neurodegeneration, it has been hypothesized that hyperphosphorylated tau may be directly toxic to the cells that harbor them. Our previous studies showed that NMNAT2 overexpression in the hippocampi of rTg4510 mice, a tauopathy model [75,76], is sufficient to reduce p-hTau burden [32]. To determine whether NMNAT2’s chaperone function is required for p-hTau clearance, we first tested NMNAT2 WT and various mutants in a HEK293-tau cell line stably expressing a doxycycline-inducible human tau40 (Fig 3A and 3B) [77]. In these cells, an increase in p-hTau is induced 48–72 h after doxycycline treatment. This p-hTau is easily detected by the PHF-1 antibody that detects p-S396/404 Tau [77,78]. Overexpression of NMNAT2-WT (*p* = 0.009), -ED (*p* = 0.013), -PM (*p* = 0.011), or HSP70 (*p* = 0.024) significantly reduced p-hTau by half, but not total, hTau in the doxycycline-induced cells (Fig 3A and 3B). However, NMNAT2-ΔCT (*p* = 0.682) or -ΔcATP (*p* = 0.763) overexpression failed to reduce p-hTau levels, suggesting that NMNAT2’s chaperone function is required to reduce p-hTau in this in vitro Tau model.

To further demonstrate that NMNAT2’s chaperone activity is required for p-hTau clearance in vivo, we examined the impact of NMNAT2-WT, -ED, -ΔcATP and GFP overexpression on p-hTau levels in the hippocampus of rTg4510 mice (Fig 3C). 1 μl of 10^9^ particles of recombinant adenoassociated virus (rAAV) carrying GFP- or HA-tagged NMNAT2-WT, -ED, -ΔcATP was injected into the hippocampi of 6-wk-old rTg4510 mice (*n* = 17), which corresponds to the age when endogenous NMNAT2 begins to decline [32]. The impact of GFP or NMNAT2-WT, -ED, -ΔcATP expression on hTau was evaluated 1-mo post injection with immunostaining (Fig 3C). The MC1-antibody that recognizes conformational changes in hTau [79] was employed to evaluate the impact of NMNAT2 and its variants on hTau processing, while an HA-antibody identified exogenous NMNAT2 or its variants (Fig 3C and 3D). The overall intensity of MC1 immunoreactivity in the CA1 region of the hippocampi was quantified, and NMNAT2-WT (*p* < 0.001), -ED (*p* < 0.001), but not -ΔcATP (*p* = 0.68) significantly reduced the levels of conformationally altered hTau recognized by the MC-1 antibody (Fig 3C and 3D). To quantify the impact of NMNAT2 overexpression on hTau processing, western blot analysis was conducted with tissue homogenates prepared from EGFP and NMNAT2-WT, -ED, -ΔcATP rAAV transduced hippocampi of rTg4510 mice one mo postinjection (Fig 3E and 3F). The levels of p-hTau were evaluated with AT8 (recognizing p-S202/205 hTau [80]) and PHF-1 antibodies. Overexpressing NMNAT2-WT (*p* < 0.001), -ED (*p* < 0.001), but not NMNAT2-ΔcATP (p = 0.59) or GFP significantly reduced p-hTau levels in rTg4510 hippocampi while total hTau levels remained unchanged (Fig 3F). These data provide compelling evidence that NMNAT2’s chaperone activity but not its enzymatic function is critical in ameliorating proteotoxic stress caused by hTau overexpression.

### NMNAT2’s Foldase Activity Requires an NMNAT2:HSP90 Complex

**Fig 4:**
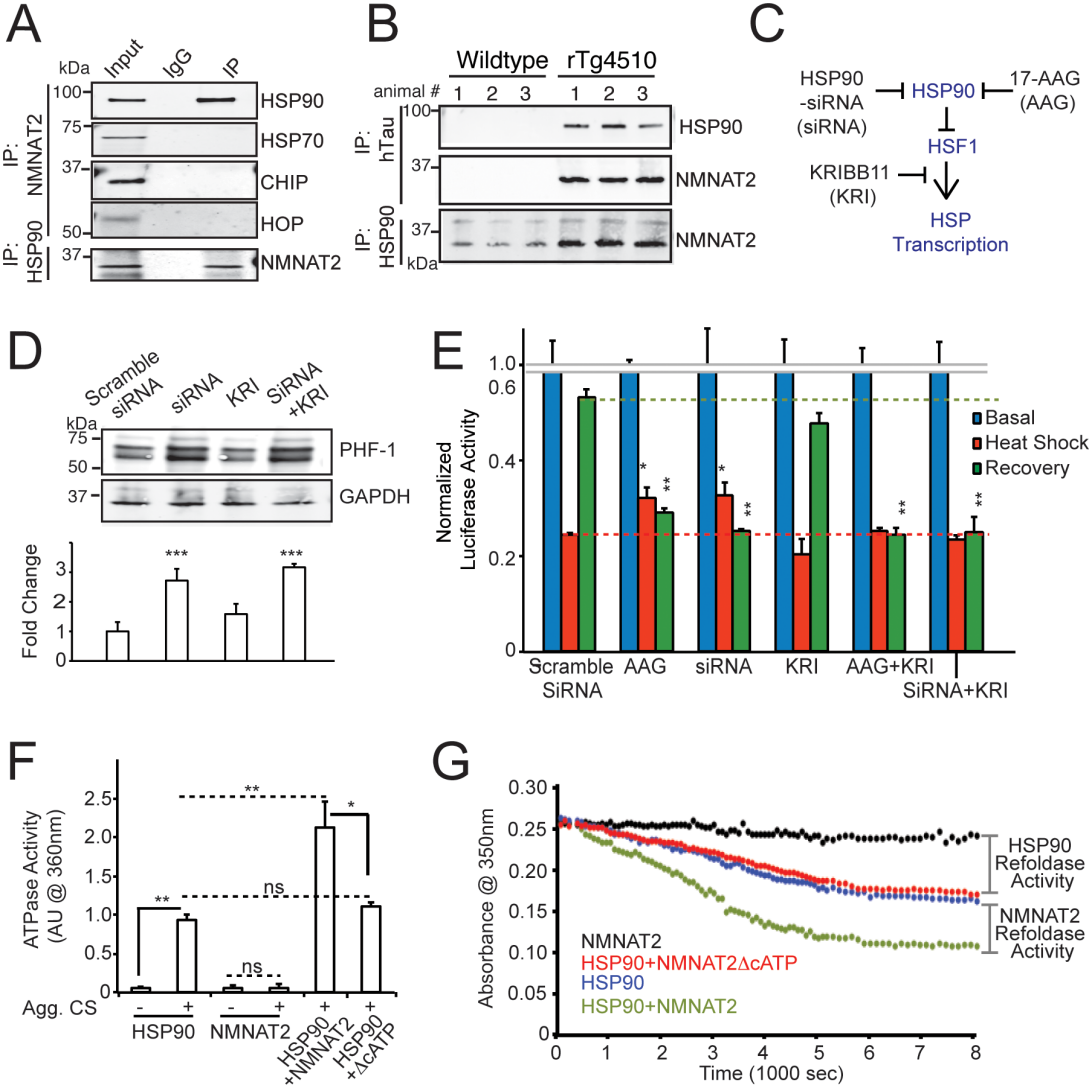
NMNAT2 complexes with HSP90 to refold aggregated proteins. (**A**) Immunoprecipitation identifies an interaction between NMNAT2 and HSP90, but not between NMNAT2 and HSP70 or HOP or CHIP in the hTau40 cell line. (**B**) NMNAT2 complexes with hTau and HSP90 in insoluble fractions of 6-mo-old rTg4510 cortex. (**C**) A simple diagram illustrating how HSP90 regulates HSF1 activity. (**D**) Inhibition of HSP90 by siRNA prevents the NMNAT2-dependent clearance of p-hTau, while inhibition of HSF1 by KRI is ineffective. Bar graph shows the summary from three independent experiments. (**E**) Summary of luciferase assay in NMNAT2-expressing cells with various treatments (*n* = 4 with triplicate). (**F**) Summary of ATPase activity of HSP90 and NMNAT2 (WT/ΔcATP) in the absence or presence of aggregated CS. (**G**) WT, but not NMNAT2-ΔcATP, can refold aggregated CS in the presence of HSP90 and ATP. Individual values for 4D, 4E, 4F, and 4G are provided in S1 Data. ns, not significant, *p < 0.05, **p < 0.001, ***p < 0.0001.

How does NMNAT2 act as a chaperone? Does NMNAT2 function together with known chaperones HSP70/90 or cochaperones HOP and CHIP [81] to reduce protein aggregates? To determine if NMNAT2 interacts with other chaperones, we conducted immunoprecipitation experiments of NMNAT2 to determine whether HSP70, HSP90, HOP, or CHIP complex with NMNAT2 in hTau40 cell lines after induction of hTau40 expression. As shown in Fig 4A, NMNAT2 complexes with HSP90 but not HSP70, HOP or CHIP in NMNAT2-transfected HEK293-tau cells, via its C-terminus (S6 Fig). Similar to NMNAT2, HSP90 is also present in the insoluble fraction prepared from human AD brains but not control brains (Fig 1E). In aged rTg4510 cortex, NMNAT2 is also present in the insoluble fraction where p-hTau and HSP90 are concentrated (S5 Fig). NMNAT2 and HSP90 not only form complexes with each other (S6 Fig) in rTg4510 cortex (Fig 4B) but also bind to hTau (Fig 4B). These interactions are likely to be evolutionarily conserved as proteomic studies in *Drosophila* also identified HSP83, the *Drosophila* HSP90 homolog, as a *d*NMNAT-interacting protein (S4 Table).

HSP90 binds to non-native polypeptides to prevent aggregate formation, mainly at the late stages of substrate folding, a function that is tightly regulated [81]. To determine whether HSP90 is required for NMNAT2’s ability to reduce protein aggregates, we reduced HSP90 levels by either using HSP90-specific siRNA or by pharmacological blockade with 17-allylamino-17-demethoxygeldanamycin (17-AAG), an HSP90-specific inhibitor [82] (Fig 4C). We first examined the impact of reducing HSP90 expression level on NMNAT2’s ability to attenuate p-hTau burden. Briefly, cells were transfected with NMNAT2-WT, and either HSP90 siRNA or scrambled siRNA 12 h prior to doxycycline induction. p-hTau abundance in these cells was examined 48 h post doxycycline induction. We found that decreasing HSP90 function severely impaired NMNAT2’s ability to reduce p-hTau (Fig 4D; *p* < 0.001for siRNA).

To determine whether NMNAT2 requires HSP90 to exert its function as a holdase or a foldase, we performed luciferase-refolding assays (Fig 2A). The cells were transfected with Luciferase and NMNAT2-WT together with scrambled or HSP90 siRNA (Fig 4E). To test the drug, the cells were treated with 500 nM 17-AAG, 24 h prior to heat shock treatment. We found that HSP90 reduction by either HSP90-siRNA (*p* < 0.001) or 17-AAG (*p* < 0.001) prevented NMNAT2’s facilitation of the recovery of aggregated luciferase, but these treatments have a minimal impact on NMNAT2’s holdase activity (Fig 4E). These data suggest that NMNAT2 requires HSP90 for its foldase activity to reduce the proteotoxic burden.

HSP90 can regulate the stress response by modulating the activity of Heat Shock Factor 1 (HSF1). During nonstressed condition, HSP90 binds to HSF1 and p23-immunophilins [83]. This binding prevents HSF1 from activating the transcription of the heat shock response genes. Upon stress or heat shock, protein aggregates sequester HSP90 [83,84], freeing HSF1 to activate transcription of the heat shock response genes [83]. Hence, reduction or inhibition of HSP90 promotes the heat shock response (Fig 4C, S7 Fig). To assess if HSF1 plays a role in the chaperoning activity of NMNAT2, we blocked HSF1 effects on HSP transcription using 10 μM KRIBB-11 (KRI) (Fig 4C) [85,86]. KRI application minimally impacted NMNAT2’s chaperone activity (*p* = 0.47, Fig 4E). Moreover, coapplication of KRI with either AAG or an siRNA for HSP90 resulted in changes that were similar to those seen with AAG or siRNA alone (*p* = 0.54, compared to siRNA alone). Consistent with the data obtained from the luciferase assay, HSF-1 inhibition had no impact on NMNAT2-mediated clearance of p-hTau (Fig 4D). These observations rule out a contribution by HSF1 to NMNAT2’s chaperone function. Taken together, our results show that NMNAT2 requires HSP90 for its foldase activity to reduce the proteotoxic burden in this hTau model.

ATPase activity is often required for molecular chaperone activity. As demonstrated above, NMNAT2’s C-terminal ATP binding site is essential for its foldase activity. Hence, we tested whether this site has ATPase activity. Unlike HSP90, recombinant NMNAT2 shows no measurable ATPase activity in the presence of denatured CS substrate (Fig 4F, S8 and S9 Figs). However, addition of HSP90 stimulated NMNAT2’s ATPase activity (*p* = 0.0002 compared to HSP90; Fig 4F, S8B Fig). The importance of the C-terminal mutant missing this ATP binding site for this ATPase activity is demonstrated by the observation that the C-terminal mutant without this ATP site lacked ATPase activity in the presence of misfolded CS and HSP90 (*p* = 0.38 compared to HSP90; Fig 4F, S9 Fig). Our findings thus suggest that a complex of NMNAT2 with HSP90 induces a conformational change that stimulates NMNAT2’s ATPase activity, allowing it to function as a foldase (Fig 4G).

### NMNAT2: HSP90 Mitigates Proteotoxicity in a SCA1 Model of PolyQ Disorder

**Fig 5:**
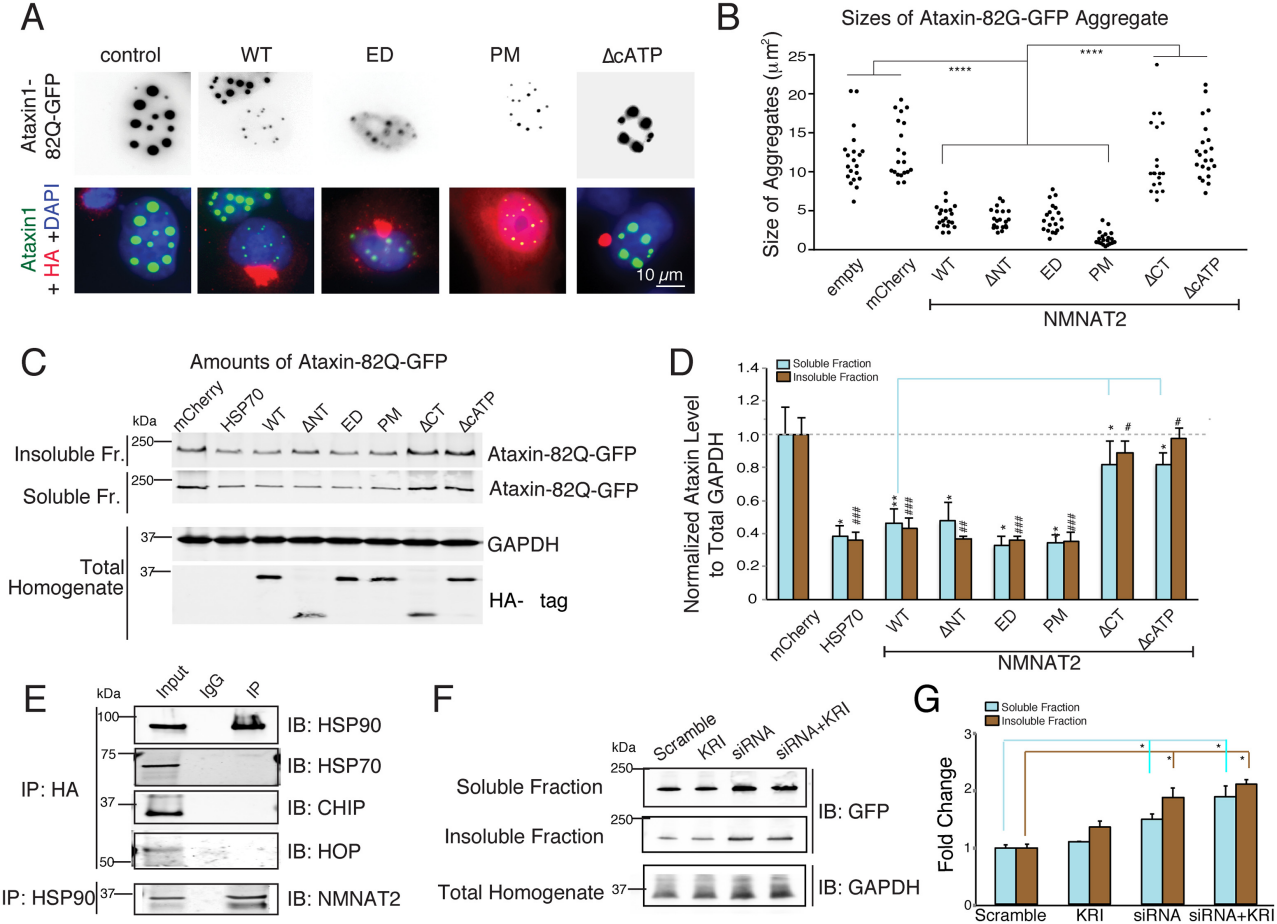
NMNAT2 reduces Ataxin1-82Q-GFP aggregates. (**A**) Immunostaining images show that cells expressing NMNAT2-WT, -ED, and -PM have fewer Ataxin1-82Q-GFP aggregates compared to untransfected cells or cells expressing mCherry, NMNAT2-ΔCT, or NMNAT2-ΔcATP (*n* = 3 independent experiments). (**B**) Summary of the sizes of individual Ataxin1-82Q-GFP aggregates present in transfected cells (**C**) Example western blot showing that expression of HSP70 and NMNAT2-WT/ΔNT/ED/PM, but not NMNAT2-ΔCT/ΔcATP have reduced Ataxin1-82Q-GFP aggregates in both the soluble and RIPA-insoluble fractions. (**D**) Summary of normalized Ataxin1-82Q-GFP levels in both the soluble and insoluble fractions of cell lysates prepared from cells expressing the indicated cDNAs (*n* = 3 independent experiments). GAPDH in total lysate was used to normalize GFP signals in the soluble and insoluble fractions. *and # indicate significant differences in soluble or insoluble Ataxin1-82Q-GFP levels between mCherry control and transfected test protein, respectively. (**E**) Immunoprecipitation identifies an interaction between NMNAT2 and HSP90, but not between NMNAT2 and HSP70 or HOP or CHIP in the Ataxin1-82Q-GFP and HA-NMNAT2 overexpressing cell line. (**F**) Western blot shows the levels of Ataxin1-82Q-GFP in both soluble and insoluble fractions upon treatment with scrambled siRNA, HSP90-siRNA (siRNA), KRI, or the combination of siRNA and KRI (*n* = 3 independent experiments). **(G)** Inhibition of HSP90 expression, but not HSF1 activity, increases Ataxin1-82Q-GFP accumulation (*n* = 3 independent experiments). Individual values for 5B, 5D, and 5G are provided in S1 Data. ns, not significant, *^/#^*p* < 0.05, **^/##^*p* < 0.001, ***^/###^*p* < 0.0001.

As *nmnat2* transcript levels are reduced in various proteinopathies (S5 Table), we examined whether NMNAT2’s chaperone function is able to reduce aggregates of polyglutamine repeat expanded Spinocerebellar Ataxia type 1 (SCA1) protein. We conducted a cell-based human Ataxin1-[82Q]-GFP aggregation assay [87], in which the polyglutamine protein forms nuclear aggregates, observed in SCA1 pathology (Fig 5). Ataxin1-[82Q]-GFP forms numerous and often large protein aggregates in nucleus and cytoplasm of Cos7 cells (Fig 5A and 5B). In the presence of NMNAT2-WT (*p* < 0.0001), -ΔNT (*p* < 0.0001), -ED (*p* < 0.0001), -PM (*p* < 0.0001), the size of these GFP aggregates are significantly smaller than the aggregates found in mCherry expressing cells, or neighboring untransfected cells (empty). However, NMNAT2-ΔCT (*p* = 0.52) or -ΔcATP (*p* = 0.631) overexpression failed to reduce the size of Ataxin1-[82Q]-GFP aggregates, again suggesting that the chaperoning activity of NMNAT2 plays a critical role in dampening the formation of protein aggregates.

To distinguish between soluble and insoluble Ataxin1-[82Q]-GFP aggregates, cells were lysed and fractionated [87]. NMNAT2 overexpression reduced the amount of both soluble (*p* = 0.0004) and insoluble (*p* < 0.0001) Ataxin1-[82Q]-GFP aggregates (Fig 5C and 5D). Again, NMNAT2’s chaperone function, but not NAD synthase activity, is required for ameliorating the proteotoxic burden in SCA-1. Furthermore, in Ataxin1-[82Q]-GFP and NMNAT2 expressing Cos7 cells, NMNAT2 also complexes with HSP90, but not with HSP70, CHIP, or HOP (Fig 5E). Knocking-down HSP90 with HSP90 siRNA also prevented the reduction of soluble or insoluble ataxin1 aggregates by NMNAT2 (Fig 5F and 5G). However, HSF-1 inhibition with KRI minimally affected NMNAT2-mediated clearance of Ataxin1-82Q-GFP aggregates. Hence, HSF1-mediated transcription does not mediate NMNAT2’s chaperone function in this model system. All the above results provide strong evidence that the NMNAT2: HSP90 complex is capable of reducing aggregated proteins in different proteinopathies (S14 Fig).

### NMNAT2 Loss-of-Function Renders Cortical Neurons More Sensitive to Stress

**Fig 6:**
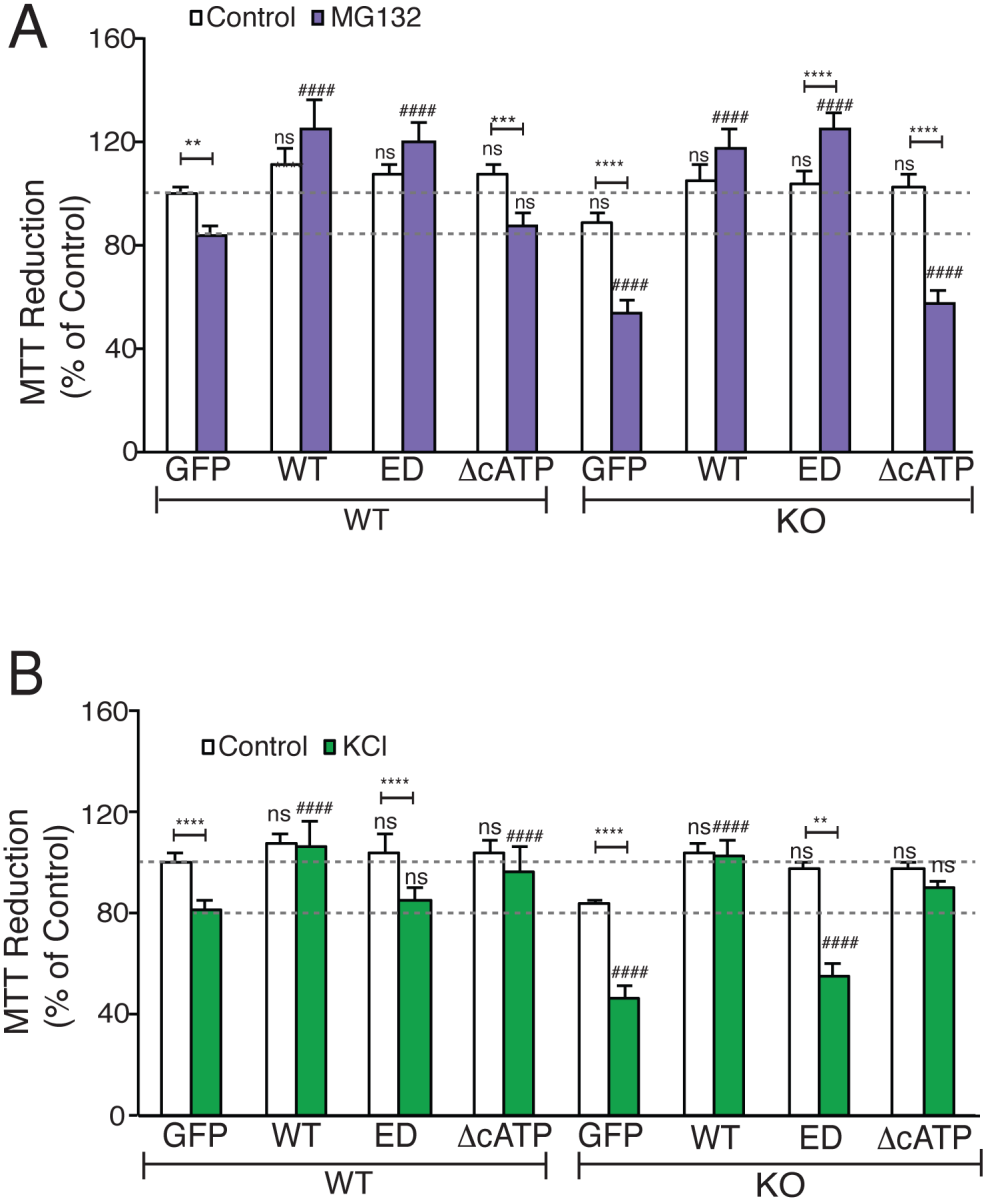
NMNAT2 is required to protect neurons against proteotoxicity and excitotoxicity. Cell viability was evaluated by the MTT reduction assay. (**A**) MTT reductions of NMNAT2 WT and KO neurons overexpressing GFP or NMNAT2-WT, -ED, or -ΔcATP after DMSO or MG132 treatment. *and # indicate significant differences in DMSO or in MG132-treated cells between GFP control and transfected test NMNAT2 construct, respectively (*n* = 3 independent experiments). (**B**) MTT reduction by DIV14 NMNAT2 WT and KO neurons overexpressing GFP or NMNAT2-WT, -ED, or -ΔcATP after DMSO or KCl treatment (*n* = 3 independent experiments). *and # indicate significant differences in DMSO or in KCl-treated cells between GFP control and transfected NMNAT2 construct, respectively. *n* = 3 with triplicates per summary. Individual values for 6A and B are provided in S1 Data. ns, not significant, *^/#^*p* < 0.05, **^/##^*p* < 0.001, ***^/###^*p* < 0.0001.

Loss of NMNAT2 in mice causes death at birth [18]. To assess NMNAT2’s function in the central nervous system (CNS), cortical neurons were prepared from NMNAT2 WT (NMNAT2+/+), HET (NMNAT2+/-), and KO (NMNAT2-/-) embryos. We quantitatively compared the viability of NMNAT2 WT, HET, and KO cortical neurons at different days-in-vitro (DIV) using the MTT (3-(4,5-dimethylthiazol-2-yl)-2,5-diphenyltetrazolium bromide) reduction assay, a colorimetric assay routinely used to estimate the number of viable cells [88]; Fig 6). We found that the viability of NMNAT2 KO neurons decreases as they mature and form numerous synapses (S10A Fig). Next, we treated DIV14 cortical neurons with 10 μM MG132, a proteosomal inhibitor, for 12 h to promote their demise via proteotoxic stress. Interestingly, loss of NMNAT2 also increases the vulnerability of DIV14 cortical neurons to proteotoxic stress (Fig 6A; *p* < 0.0001), suggesting that NMNAT2 deletion renders neurons sensitive to proteosomal stress. To determine whether NMNAT2’s chaperone function is required for its ability to protect cortical neurons against MG132-induced proteotoxicity, we transduced NMNAT2 KO cortical neurons with a lentiviral vector (LV) carrying NMNAT2 WT or various mutants at DIV2. At DIV14, the viability of these neurons was assessed by the MTT reduction assay after MG132 treatment. Overexpression of NMNAT2-WT (*p* = 0.0024), or -ED (*p* = 0.0013), but not NMNAT2-ΔcATP (*p* = 0.784), significantly increased the viability of NMNAT2 KO neurons post MG132 treatment (Fig 6A, S11 Fig). Interestingly, in WT neurons, NMNAT2 overexpression completely mitigated the toxic impact of protein stress (Fig 6A). Such protection by NMNAT2 depends on its chaperone but not enzymatic function. Hence, the chaperone function of NMNAT2 is required to protect cortical neurons against MG132 induced proteotoxicity, whereas its NAD synthesis activity is dispensable.

In *Drosophila* photoreceptors, dNMNAT protects neurons against activity-related insults [1]. Here, we found that upon massive glutamate release triggered by KCl treatment (30 mM for 24 h), the viability of NMNAT2 KO neurons was significantly decreased even at DIV10, while the viability of NMNAT2 WT and HET neurons was not affected by KCl treatment (Fig 6B, S10B and S11A Figs). At DIV20, both NMNAT2 HET (*p* < 0.01) and KO (*p* < 0.0001), neurons are significantly more sensitive to KCl-triggered excitotoxicity compared to NMNAT2 WT neurons (S10B Fig). Overexpression of NMNAT2-WT (*p* < 0.0001), -ΔcATP (*p* < 0.0001), but not NMNAT2-ED (*p* = 0.162), in NMNAT2 KO neurons significantly restored their resistance to KCl-induced cell death (Fig 6B, S11B Fig). Similarly, NMNAT2 overexpression in WT neurons completely prevented the excitotoxicity and this protection required NMNAT2’s enzymatic activity. Thus, cortical neurons require NMNAT2’s enzymatic but not chaperone function to protect them against excitoxicity. Taken together, both the enzymatic and chaperone activity of NMNAT2 are important to maintain neuronal health in a context-dependent manner.

### NMNAT2 May Play a Role in Synaptic Maintenance

**Fig 7:**
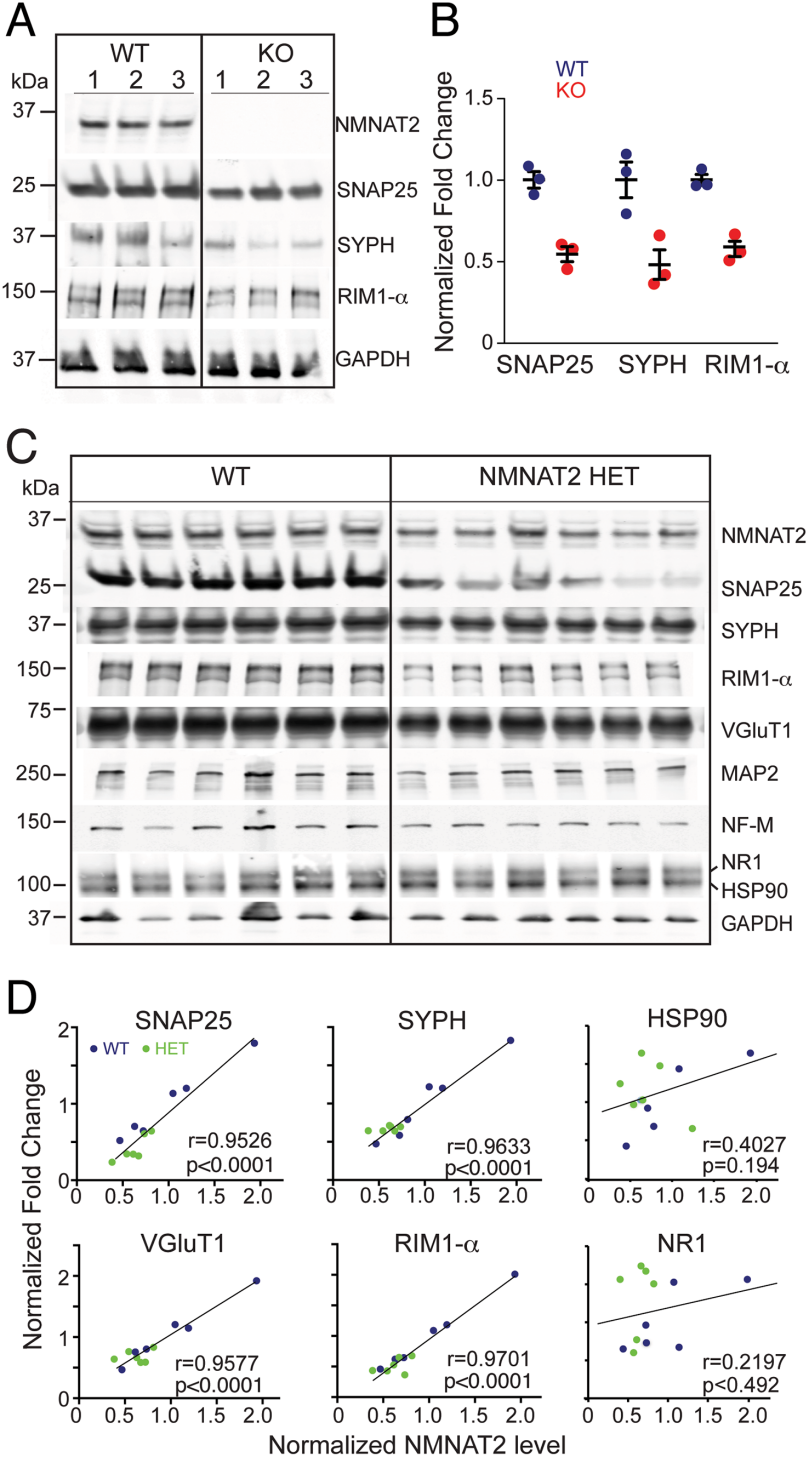
NMNAT2 abundance is positively correlated to the levels of synaptic proteins. (**A–B**) Levels of synaptic proteins analyzed by western blotting in DIV10 WT, HET and KO cortical neurons. *n* = 3 independent experiments for each genotypes. (**C–D**) Western analysis for the abundance of synaptic proteins in the hippocampi of 8-mo-old NMNAT2 HET and WT (*n* = 6 per genotype). The regression lines show the relationships between NMNAT2 and SNAP25, SYPH, VGluT1, RIM1α, HSP90, NR1. Protein levels were normalized to GAPDH. Individual values for 7B and 7D are provided in S1 Data.

Synapses are often the first structures lost in neurodegenerative disease [89]. Vertebrate NMNAT2 has been detected in synaptic membranes and vesicles of cortical neurons [17,24] suggesting a role for NMNAT2 in synapse maintenance. Immunostaining of DIV10 cultured neurons reveals a decrease of presynaptic proteins such as VGluT1 and synaptophysin (SYPH) in NMNAT2 HET neurons when compared to WT (S12 Fig. Almost no VGluT1 or SYPH signals are seen in the axons of NMNAT2 KO neurons. Loss of NMNAT2 significantly reduces levels of synaptic vesicle proteins such as SNAP25 (*p* = 0.0071) and SYPH (*p* = 0.032) as well as the active zone protein RIM1α (Fig 7A; *p* = 0.0028). In 8-mo-old NMNAT2 WT and HET brains, NMNAT2 protein levels are variable (Fig 7B and 7C). Interestingly, the abundance of presynaptic proteins like SYPH (*p* < 0.0001, r = 0.9633), VGluT1 (*p* < 0.0001, r = 0.9577), SNAP25 (*p* < 0.0001, r = 0.9526) and RIM1α (*p* < 0.0001, r = 0.9701) are positively correlated with NMNAT2 levels (Fig 7C). In addition, the levels of neurofilament (*p* = 0.033, r = 0.6159) and microtubule binding protein MAP2 (*p* = 0.0067, r = 0.7325) are also significantly correlated to NMNAT2 levels, whereas levels of HSP90 and N-methyl-D-aspartate receptor 1 (NR1) were not significantly related. Taken together, our data indicate that NMNAT2 plays a significant role in synaptic maintenance.

## Discussion

Here, we provide compelling evidence that NMNAT2 acts as a potent chaperone for denatured luciferase, p-hTau, and ataxin1-82Q-GFP. NMNAT2’s C-terminal ATP site is required for its chaperone activity but not for its NAD synthesis activity. The ED (NAD synthesis incompetent) NMNAT2 mutant has comparable chaperone activity to wildtype NMNAT2 in restoring luciferase activity as well as in reducing p-Tau and ataxin1-82Q-GFP. In rTg4510 mice, both WT- and ED-, but not chaperone-compromized-NMNAT2, were effective in reducing the abundance of both p-hTau and pathological hTau. This finding implicates an in vivo requirement of NMNAT2’s chaperone activity in p-hTau clearance. Its partial loss may therefore promote neurodegeneration in AD and other neurodegenerative diseases, whereas increased levels of NMNAT2 may protect against the demise of neurons.

NMNAT2 exerts both a holdase function, whereby it binds to aggregation-prone proteins to reduce their aggregation, and a foldase function, whereby it binds to and refolds aggregated or conformationally abnormal proteins. Using a CS aggregation assay, we found that NMNAT2 alone can act as holdase, while it requires HSP90 to refold CS aggregates. Importantly, the interaction with HSP90 activates NMNAT2’s C-terminal ATPase activity when protein aggregates are present. This C-terminus ATP binding site is required for NMNAT2’s foldase activity. It is likely that the ATPase activity triggered by HSP90 binding is required for NMNAT2 to refold its client proteins. Independently, a *Drosophila* proteomic screen also identified the HSP90 homolog, HSP83, as a dNMNAT interacting partner, suggesting the NMNAT2 and HSP90 interaction is conserved during evolution. In addition to hTau, HSP90 is also required for NMNAT2 to reduce CS aggregation as well as refolding of denatured luciferase and Ataxin1-polyQ aggregates. The conservation of such interactions across diverse systems attests to a broad role for the HSP90:NMNAT2 complex in mitigating proteotoxic aggregation.

Previous studies using nerve injury models have proposed that NMNAT2 supplies NAD to maintain axonal integrity [15–17,90]. Here, we found that deleting NMNAT2 in cortical neurons renders them sensitive to both proteotoxic stress and excitotoxicity triggered by sustained neuronal depolarization. In wildtype neurons, NMNAT2 overexpression has no impact on baseline cell viability but strengthens neuronal defense mechanisms to combat neural insults triggered by excessive protein stress or neurotransmission. Interestingly, the chaperone function of NMNAT2 is required to counter protein stress while the enzymatic function of NMNAT2 provides protection against excitotoxicity. The restoration of NMNAT2 KO neurons’ resistance to activity induced insults by over expression of enzymatically competent NMNAT2 reveals a similar context-dependent protection mechanism.

It remains to be determined how NMNAT2 switches between these two functional modes to protect against these distinct insults. Using Cos7 cells, we demonstrate that NMNAT2 only complexes with HSP90 upon protein stress (S13 Fig) despite both proteins being highly expressed. The observation of increased NMNAT2:HSP90 complex formation in the hippocampi of rTg4510 mice compared to wildtype mice suggests that protein stress triggered NMNAT2:HSP90 complex formation also occurs in vivo. Interestingly, NMNAT2’s ATPase activity is only activated when both HSP90 and protein aggregates are present. Taken together, we propose that NMNAT2 forms complexes with different partners depending on the nature of the insult and complex formation allows it to switch modes of actions to offer neuroprotection.

The localization of NMNAT2 in synaptosomal fractions prepared from whole mouse brains suggests the presence of NMNAT2 in synapses [24]. In *Drosophila*, dNMNAT maintains synaptic structure by stabilizing the active zone protein Bruchpilot [91], and photoreceptors that lack dNMNAT are sensitive to light or neural activity [1]. Loss of NMNAT2 significantly reduces the levels of presynaptic proteins. Previous studies have demonstrated a critical role of NMNAT2 in neurite outgrowth [18,19]. The reduction in axon number and length can account for the reduction of presynaptic proteins we detected in NMNAT2 KO neurons. However, immunostaining reveals reduced immunoreactivity for synaptic vesicle proteins VGluT1 and synaptophysin in NMNAT2 KO axons (S12 Fig). This suggests that NMNAT2 is required not only for axonal outgrowth but also for maintaining synaptic proteins in axonal arbors. The finding of a positive correlation between NMNAT2 levels and presynaptic proteins in adult mice provide additional support for the role of NMNAT2 in maintaining synaptic integrity. The dual-function of NMNAT2 makes it a very potent neuronal maintenance factor; it can provide sufficient local NAD to maintain neurons after high frequency neurotransmission and can also act as a chaperone to reduce proteinopathies. Such dual protection is likely to be important in preventing synaptic loss and preserve cognitive function during aging.

Our studies of NMNAT2 transcript levels, AD pathology, and cognitive performance in the human cohort studies represent associations, and on their own, do not establish causality. It is interesting that NMNAT2 but not NMNAT1 levels are reduced in AD brains. The shift of NMNAT2 solubility to an insoluble compartment in AD brains and its partitioning with hyperphosphorylated hTau, provides strong support for NMNAT2’s role as a molecular chaperone in the human brain. Due to NMNAT2’s short half-life [15,17], the appropriate level of NMNAT2 is likely to be actively regulated to ensure optimal neuronal function. In rTg4510 mice, NMNAT2 levels are reduced prior to neurodegeneration, a reduction due in part from decreased CREB-mediated *nmnat2* transcription [32]. Decreased CREB activity has been reported in human AD brains and mouse AD models [92]. Thus, it is plausible in human that reduced CREB activity results in a decrease in NMNAT2 and synaptic loss, culminating in dementia. Deleting NMNAT2 function in mouse cortical neurons decreases presynaptic markers and increases sensitivity to excitotoxicity and protein stress. NMNAT2’s enzymatic activity is required to reduce excitotoxicity upon excessive neurotransmission, while it works together with HSP90 to reduce protein stress through its chaperone function. Elucidating how *nmnat2* levels are regulated may help to reveal preventive therapies to protect neurons from *nmnat2* down-regulation and cognitive decline. NMNAT2’s chaperone function is a promising therapeutic target to protect against CNS proteinopathies.

## Materials and Methods

### Study Approval

The ROS (ORA#91020181) and MAP (ORA#86121802) studies were conducted in accordance with the latest version of the Declaration of Helsinki and were approved by the Institutional Review Board of Rush University Medical Center. Written informed consent was obtained from all subjects, followed by an Anatomic Gift Act for organ donation. For human brain biochemistry, frozen cortical tissue was obtained from the Oregon Brain Bank at Oregon Health and Science University in Portland, OR. Tissue use conformed to institutional review board-approved protocols. Animal protocol 4307, 14–031 were approved by the IACUC at Baylor College of Medicine and Indiana University, respectively.

### Religious Orders Study (ROS) and Rush Memory and Aging Project (MAP)

The ROS cohort, established in 1994, consists of more than 1,100 Catholic priests, nuns, and brothers from 40 groups in 12 states who were at least 55 y of age and free of known dementia at the time of enrollment. The MAP cohort, established in 1997, consists of more than 1,600 men and women primarily from retirement facilities in the Chicago area who were at least 53 y of age and free of known dementia at the time of enrollment. All participants in ROS and MAP signed an informed consent agreeing to annual detailed clinical evaluations and cognitive tests, and the rate of follow-up exceeds 90%. Similarly, participants in both cohorts signed an Anatomical Gift Act donating their brains at the time of death. The mean for the interval between last study evaluation and death is 8.2 mo. The overall autopsy rate exceeds 85%. All aspects of these studies were approved by the Institutional Review Boards of Rush University Medical Center and Partners Healthcare. More detailed information regarding the two cohorts can be found in previously published literature [52,53]. The global cognitive performance score has been previously described and was determined based on 17 cognitive tests, measuring 5 domains of cognitive function (episodic memory, visuospatial ability, perceptual speed, semantic memory, and working memory), and collected at annual evaluations proximate to death [55]. Results of each test were converted into a Z score using the mean and SD from baseline evaluations of all participants and then averaged to compute the composite measure of global cognition. For descriptive purposes and for the subgroup analysis presented in both S1 and S2 Tables, and Fig 1, the clinical diagnoses of dementia and AD were made following National Institute of Neurological and Communicative Disorders and Stroke–Alzheimer’s Disease and Related Disorders Association recommendations [93,94]. Mild cognitive impairment (MCI) referred to those individuals rated as cognitively impaired by the neuropsychologist but not demented by the examining physician, as previously described [95]. The quantitative global AD pathology measure has been extensively described in prior publications [56]. Bielschowsky silver stain was used to visualize and count neuritic plaques and neurofibrillary tangles on representative sections from midfrontal, middle temporal, inferior parietal, and entorhinal cortices, and the hippocampal CA1 sector. Raw counts were divided by the population standard deviation of the region-specific counts, and the scaled counts were averaged over the five brain regions to create a global summary measure. RNA-seq data was generated using postmortem brain tissue (dorsolateral prefrontal cortex) from ROS/MAP subjects. The RNA was extracted from the tissue after the quality control evaluation based on RIN score [96] or RNA were extracted first, and then RIN was used to filter out low-quality samples. The library was sequenced using Illumina Hi-Seq with 101 bp reads and 4-plex pooling. All the paired-end reads were mapped using RSEM alignment using Bowtie as the aligner [97] using the human genome transcriptomic database from Ensemble (http://www.ensembl.org). The Fragment Per Kilobase Per Million (FPKM) was calculated for the *nmnat1* or *nmnat2*, and these values were regarded as expression quantity for each gene and its isoform for further analysis. Linear regression was used to evaluate the relationship between transcript levels (*nmnat1* or *nmnat2*) and either global cognition or global AD pathology summary score. Since the scaled outcome of AD pathologic burden was right skewed, we applied square root transformation prior to the analyses. All analyses were adjusted for age at death, gender, postmortem interval, and RIN score. *p* < 0.0125 was considered significant after adjusting for multiple hypothesis testing (α = 0.05 divided by 4 [2 transcripts + 2 outcomes]. Path analysis was used to evaluate structural models linking *nmnat2* mRNA expression, global AD pathology, and global cognition proximate to death. Standardized path coefficients along individual pathways were used to assess the total direct and indirect effects of *nmnat2* expression on cognition. All tests were performed using SAS software, version 9.3 (SAS Institute, Cary, NC) and Mplus, version 7.0 (Muthen & Muthen, 1998–2012).

### Human Brain Samples

Frozen cortical tissue was obtained from the Oregon Brain Bank at Oregon Health and Science University (OHSU) in Portland, OR. These subjects were enrolled and clinically evaluated at the NIH-sponsored Layton Aging and AD Center (ADC) at OHSU. Every subject received annual neurological and neuropsychological evaluations, with a clinical dementia rating (CDR) assigned by an experienced clinician. The AD subjects were diagnosed according to the National Institute for Neurological and Communicative Disorders and Stroke-Alzheimer’s Disease and Related Disorder Association diagnostic criteria for clinical AD, followed by neuropathologic confirmation at autopsy. Neuropathologic assessment conformed to National Institute on Aging-Reagan consensus criteria. All brain tissue was examined by a neuropathologist for neurodegenerative pathology including neurofibrillary tangles and neuritic plaques and assigned an amyloid score based on the deposition of amyloid plaques in the brain (0, no plaques; 1, sparse plaques; 2, moderate plaques; 3, dense plaques), and a Braak stage (0–6; with 6 being the most severe) indicative of the level and location of hyperphosphorylated tau tangles [98]. In addition to the pathological information detailed above, demographical data including age, sex, and MMSE score for each case were received along with the frozen tissue (S3 Table).

### Quantitative Real-Time PCR

For each sample, RNA concentration was determined by spectrophotometry at 260 nm. Two micrograms of RNA were used for the reverse transcription reaction with the High Capacity RNA to cDNA kit (Applied Biosystems, Carlsbad, CA). Quantification of *nmnat2* and *nmnat1* mRNA was performed using a CFX Touch Realtime PCR System (BioRad). The housekeeping genes, *18s* and *gapdh*, were used as internal controls to normalize mRNA expression. Amplification mix (25 μl) consisted of 0.1 μg cDNA, 12.5 μl TaqMan Universal PCR Master Mix (Applied Biosystems) and 1 μl TaqMan Gene Expression Assay (Hs00322752_m1 *nmnat2*, Hs00276702_m1 *nmnat1*, Hs99999901_s1 *18s*, Hs02758991_g1 *gapdh*, Mm99999915_g1). Samples were amplified by a PCR program of 40 cycles of 10 s at 95°C, 15 s at 55°C, and 1 min at 72°C. The Ct value was defined as the number of cycles required for the fluorescence to exceed the detection threshold, and the data were analyzed using the 2^−ΔΔCt^ method to quantitatively assess relative changes in gene expression [99]. Briefly, the raw Ct values were averaged for samples per group (AD, NDAN, and age-matched controls) for *nmnat1*, *nmnat2*, *gapdh*, *and 18s*. ΔCt values were calculated by subtracting the geometric means of *gapdh* and *18s* Ct value from that of *nmnat1* and *nmnat2*. The relative expression of *nmnat1* and *nmnat2* in AD and NDAN samples compared with controls was obtained by calculating the ΔΔCt value for each group by subtracting the average ΔCt value of control *nmnat1*/*nmnat2* from AD/NDAN average ΔCt. The relative fold change of AD/NDAN groups to control was expressed as 2^−ΔΔCt^.

### Human Tissue RNA Extraction

Total RNA was extracted from human brain samples using Trizol reagent (Life Technology, Grand Island, NY). 0.5 mL of 100% isopropanol was added to the aqueous phase, per 1 mL of TRIzol Reagent used for homogenization and the samples were incubated at room temperature for 10 minutes before centrifuging at 12,000 × g for 10 minutes at 4°C. RNA was purified from the extracts using RNeasy Lipid Tissue Mini kit (Qiagen, Gaithersburg, MD) according to manufacturer’s protocol. RNA concentration and purity were determined using a Nanodrop spectrophotometer.

### Human Tissue Protein Extraction

Human brain samples were prepared for protein extraction using the differential fractionation method previously described [61,62]. This method relies on the use of increasingly stringent detergents that allow for soluble and peripheral membrane proteins to be extracted first, followed by extraction of membrane-bound proteins, and finally the most insoluble proteins are extracted into a 2% SDS buffer. Briefly, brain tissue was collected and frozen immediately with liquid nitrogen. Prior to extraction, the frozen brain tissue was weighed and homogenized in 15 volumes of ice-cold TBS (50 mM Tris-HCl, pH 7.4 and 150 mM NaCl) containing protease and phosphatase inhibitors (Roche, Branford, CT). The samples were centrifuged at 175,000 x g for 1 h at 4°C and the supernatants collected as the TBS fraction. The pellets were resuspended in ice-cold TBS buffer, spun at 175,000 x g for 1 h at 4°C, and the supernatants were discarded. Ice-cold TBS (pH 7.4) containing protease and phosphatase inhibitors and 1% Triton X-100 (TBS-X) were added to the pellet and the suspension homogenized. The samples were centrifuged at 170,000 x g for 1 h at 4°C and the supernatants collected as the TBS-TritonX fraction (soluble fraction). During the next myelin flotation phase, the pellet from the previous step was washed in TBS-TritonX buffer with 30% sucrose at room temperature for 1 h (with rotational shaking). This fraction was centrifuged at 175,000 x g for 1 h at 4°C, and the supernatants were discarded. The final pellet containing the insoluble material was re-suspended in 2% SDS buffer (insoluble fraction). Protein concentrations were determined using the Bradford Assay (BioRad, Hercules, CA).

Upon measurement of total protein concentrations, this extraction scheme applied to control brains yields 32% of total proteins in the TBS fraction, 65% in the TBS-TritonX fraction, and around 2% in the insoluble fraction. In comparison, in AD brains, around 26% of total protein is present in the TBS fraction, 68% in TBS-TritonX fraction and roughly 5% in the insoluble fraction, reflecting the higher level of insoluble protein present in AD brains.

### Western Blotting

For analysis of protein expression, 10 μg of total protein lysate per sample were resolved by sodium dodecyl sulfate polyacrylamide gel electrophoresis and transferred onto a nitrocellulose membrane, and probed with antibodies against the HA-tag (1/5,000; Cell Signaling, Danvers, MA), ß-actin (1/4,000; Sigma), hTau (1/1,000; DAKO, Glostrup, Denmark), GFP (1/1,000, Invitrogen), GAPDH (1/5000, Millipore, Temecula, CA), PHF-1 (1/1,000, gift from Dr. Peter Davies), AT8 (P1/1,000 Pierce/Thermofisher, Waltham, MA), Neurofilament-M (1/2,000, Millipore, Temecula, CA), HSP70 (1/1,000, Abcam, Cambridge, MA), HSP90 (1/1,000, Novus Biological, Littleton, CO), CHIP (1/1,000, Novus Biological, Littleton, CO), HOP (1/1,000, Novus Biological, Littleton, CO). Western blot analysis was performed with infrared dye conjugated secondary antibodies, IR700 and IR800 (1/10,000, LI-COR Biosciences, Lincoln, NE). Blots were imaged and processed on an Odyssey Infrared Imaging System. Densitometry analysis was performed on all the blots using ImageJ software (NIH). Data are represented as means ± SEM.

To study changes in neuronal proteins in aged NMNAT2-HET and wild type littermate brains, the cortex and hippocampus were dissected out and homogenized in Syn-PER Synaptic Protein Extraction buffer (ThermoFisher Scientific, GrandIsland, NY). Part of the total homogenate from each brain was stored, while the rest was used for synaptic fractionation, to enrich for synaptic proteins, using manufacturer’s protocol. Protein concentrations were calculated using Bradford Assay (BioRad). For analysis of protein expression, 10 μg of total protein lysate per sample were resolved by sodium dodecyl sulfate polyacrylamide gel electrophoresis, transferred onto a nitrocellulose membrane, and probed with antibodies against Synaptophysin (1/4,000, Millipore, Temecula, CA), SNAP-25 (1/1,000, Synaptic Systems, GmbH, Germany), Neurofilament (1/1,000, Millipore, Temecula, CA), VGlut2 (1/1,000, Synaptic Systems, GmbH, Germany), GAPDH (1/5,000, Millipore, Temecula, CA).

### Plasmid Cloning

pCMV-HSP70, pCMV-NMNAT1, pCMV-NMNAT2, pCMV-NMNAT3 and pCMV-cytLuciferase were gifts by Dr. R. Grace Zhai (University of Miami). pEGFP-hAtaxin-1[82Q] was a gift by Dr. Huda Y. Zoghbi (Baylor College of Medicine). Site-directed mutagenesis was performed on the pCAG-HA-NMNAT2 backbone using a Quik-ChangeII Site-Directed Mutagenesis kit (Agilent, Santa Clara CA). The sequences of primers used for mutagenesis are listed below. NMNAT2^W92G^-forward: 5’-TGC TAC CAG GAC ACC GGT CAG ACG ACC TGC AGC-3’. NMNAT2^W92G^-reverse: 5’-gct gca ggt cgt ctg acc ggt gtc ctg gta gca-3’. NMNAT2^ΔNT^-forward, 5’- gga aaa cag ggc ctc gtg tca agc cgg-3’ NMNAT2^ΔNT^-reverse. 5’- cgt ggg tct tgg tgg tct cgg t-3’. NMNAT2^ΔCT^-forward, 5’- ctg tac atc aat gcc tcc ggc tag-3’. NMNAT2^ΔCT^-reverse, 5’- cac tac cac aca gca gca gga tcc gta gct c-3’. NMNAT2^ΔcATP^-forward, 5’-gtg gat tac ctg tcc cag ccg gtc atc gac tac-3’. NMNAT2^ΔcATP^-reverse, 5’-ctt ggt tga gct gac aac aga cat ggg-3’. NMNAT2^C164S/C165S^-forward, 5'-aag cct cag ccg gat ctc ctc tgt ccg ccc-3'. NMNAT2^C164S/C165S^-reverse, 5'-ggg cgg aca gag gag atc cgg ctg agg ctt-3'. For rAAV generations, cDNAs carrying NMNAT2-WT, -ED, -ΔcATP were respectively cloned into BamHI or NotI sites 5’ to the expression cassette-IRES-EGFP driven by the elongation factor-1 (EF-1) promoter within the AV4-EF1-GFP shuttle plasmid as described [32].

### CS Aggregation Assay

The CS aggregation assay was performed as described previously [67]. Substrate CS (Sigma, St. Louis, MO) was desalted and mixed with either egg white lysozyme (Sigma), NMNAT1/2/3 (R&D Systems, Minneapolis, MN), or HSP70 (R&D Systems, Minneapolis, MN) at varying concentrations in HEPES (pH 7.4) buffer for 30 min. To generate recombinant NMNAT2 proteins, the coding sequences were cloned into pET28b vector for bacterial expressions. The recombinant proteins of NMNAT2 and its variants were prepared by the MD Anderson Proteomic Core. Aggregation of denatured CS was initiated at 43°C and was monitored as Raleigh scattering absorbance at 360 nm as a function of time. A FluoStar Optima plate reader (BMG Labtech, Cary, NC) was used for absorbance measurements. The relative chaperone activity of NMNAT was calculated as the scattering of CS aggregates with time versus NMNAT concentration.

### CS Refolding Assay

The refolding assay of chemically denatured CS was performed as described previously [100]. Briefly, the concentration of desalted CS was measured at A_280_ (for CS, A_280_(0.1%) = 1.78). 1 μM CS was denatured in 6 M guanidine hydrochloride, 1 M Tris-HCl (pH = 8) and 50 mM dithiothreitol (DTT) for 1.5 hours at 25°C. Renaturation of aggregated CS was initiated by adding 0.5 M ATP and 1 μM of respective chaperones and studied by measuring light scattering at 360 nm as a function of time. A FluoStar Optima plate reader (BMG Labtech, Cary, NC) was used for absorbance measurements.

### Luciferase Aggregation Assay

The luciferase aggregation assay was performed as described [101,102], with a few modifications. Cos7 cells were transfected with pCMV-*cyt*Luciferase and one of the following plasmids: pCMV-HSP70, pCMV-NMNAT1, pCMV-NMNAT3, pCAG-mCherry, HA-NMNAT2, HA-NMNAT2^W92G^(ED), HA-NMNAT2^C164S/C165S^ (PM), HA-NMNAT2^ΔNT^, HA-NMNAT2^ΔCT^, or HA-NMNAT2^ΔcATP^, using Lipofectamine (Invitrogen, Grand Island, NY). 48 h post-transfection, the cells were treated with the protein synthesis inhibitor cycloheximide (50 ug/mL for 3 h). One batch of cells was lysed immediately with luciferase lysis buffer (Promega, Madison, WI), while two similar batches were heat-shocked at 42°C for 15 min (which induced efficient unfolding of luciferase without killing the cells). Second batch of cells was lysed immediately after heat shock, while the final batch was allowed to recover at 37°C for 3 h. Luciferase activity was measured with the Luciferase Assay System (Promega).

### NAD/NADH Enzymatic Assay

NMNAT enzyme activity was measured using Amplite Colorimetric Total NAD and NADH Assay Kit (AAT Bioquest, Sunnyvale, CA). Briefly, Cos7 cells overexpressing different NMNAT isoforms and mutants were lysed 48 h post transfection using manufacturer’s protocol. For measuring NAD levels from hippocampi, tissue was extracted in the lysis buffer provided in the kit and NAD was measured using 50 ug total protein per reaction. NAD/NADH levels were measured using supplied standards using an absorbance microplate reader at ~576 nm.

### ATPase Activity Assay

The ATPase activity of NMNAT2 variants and HSP90 was measured with or without aggregated CS using the EnzCheck Phosphate Assay kit (Life Technologies, Grand Island, NY). Briefly, this spectrophotometric microplate-based assay detects a change in absorbance from 330 nm to 360 nm, when the 2-amino-6-mercapto-7-methylpurine riboside (MESG) substrate is converted by the purine nucleoside phosphorylase (PNP) enzyme to the ribose MESG product, in the presence of inorganic phosphate. The reactions were performed in a 96-well plate, in the presence of 500 mM ATP and either 1 mM NMNAT2-WT, -ΔcATP or HSP90, in the absence or presence of denatured CS. Measurements were made after 5 min of incubation according to the manufacturer’s protocol.

### In Vitro p-hTau Assay

Inducible tau stable Trex-293 cell lines expressing human 2N4R tau (hTau40) were a gift from Dr. Jeff Kuret [77]. To maintain stably-transfected cells containing the Tau transgene, these were cultured in selective medium containing 500 μg/ml G418 (Sigma). Cells were transfected with pCMV-mCherry, HSP70, HA-NMNAT2, HA-NMNAT2-ED, HA-NMNAT2^-^PM, HA-NMNAT2-ΔNT, HA-NMNAT2-ΔCT or HA-NMNAT2-ΔcATP using Lipofectamine. 6 h post transfection, Tau expression was induced by adding 1 μg/mL tetracycline (Sigma) to cell culture media. 48 h postinduction, the cells were lysed in RIPA buffer to extract total protein, which was assayed for PHF-1 levels using western blotting.

### Cos7 Cell Line Experiments

HA-NMNAT2 was expressed in Cos7 cells using lipofectamine. 48 h post transfection, cells were treated with DMSO or 10 μM MG132 for 12 h. Cells were lysed in RIPA buffer and protein concentration assayed using the Bradford method. 1 mg total protein was used as the starting material for immunoprecipitation with HA-antibody or HSP90 antibody. Mouse IgG was used as a negative control in the experiment. Immunoprecipitates were probed with specific antibodies to reveal protein–protein interactions.

### Mice

The generation and genotyping of rTg(tauP301L)4510 (rTg4510) and NMNAT2-Blad mice have been described previously [18,76]. rTg4510 mice over-express the P301L mutation in 4R0N human tau associated with FTDP-17 [76]. NMNAT2 null mutation was generated by transposon-mediated gene-trap mutagenesis [18]. Animal housing and use were in compliance with the NIH Guidelines for the Care and Use of Laboratory Animals and were approved by the institutional animal care committee at Baylor College of Medicine and Indiana University.

### Stereotactic Injection of Recombinant Adeno-associated Virus Carrying GFP or NMNAT2 or NMNAT2 Variants into Adult Mice

To quantitatively compare the in vivo efficacy of NMNAT2 and its variants in reducing toxic tau species, we overexpressed GFP, and NMNAT2-WT, -ED, -ΔcATP in the hippocampi of 6-wk-old rTg4510 mice through rAAV. Serotype 6 rAAVs carrying cDNA’s of NMNAT2-WT, -ED, or -ΔcATP were prepared by cloning into the expression cassette-IRES-EGFP driven by the EF-1 promoter within the AV4-EF1-GFP shuttle as described [32]. GFP-rAAV was also generated as control rAAV. 1 μl rAAV-NMNAT2-WT, -ED, -ΔcATP, or 1 μl of rAAV-EGFP (10^8^ particles per μl) was injected into the CA1/dentate gyrus area of the hippocampus in one hemisphere (control mice: *n* = 6 for GFP, *n* = 2 per NMNAT2-WT, -ED, and ΔcATP; rTg4510 mice: *n* = 6 for GFP, *n* = 6 for NMNAT2-WT, *n* = 5 for -ED, and *n* = 6 for ΔcATP) at the following coordinates measured from bregma: A: 2 mm, L: 1–1.5 mm, V: 1.2 mm (tooth bar set at zero). The efficacy of these rAAVs on toxic tau clearance were quantitatively evaluated with immunostaining and western blotting using the tissue prepared from GFP-AAV and NMNAT2 or NMNAT2 variants-AAV-transduced hippocampi of rTg4510 mice at 1 mo postinjection.

### Immunohistochemistry and MC1 Quantification in rTg4510 Hippocampus

One month after rAAV injections, rTg4510 and control mice were deeply anesthetized, transcardially perfused with PBS, followed by 4% Paraformaldehyde (PFA) solution in PBS, after which the brains were removed and postfixed overnight in 4% PFA. The brains were serially sectioned in the coronal plane into 50 μm thick sections using a Leica VT-1000 vibrating microtome (Leica Microsystems, Bannockburn, IL). Brain slices containing comparative complete hippocampal morphology were selected and stained with MC-1 antibody (recognize pathological conformation of hTau, a generous gift from Peter Davies, Feinstein Institute for Medical Research, Manhasset, NY; 1:500), HA-antibody (1:1,000; Cell Signaling, Danvers, MA) and DAPI (nuclear stain). For quantitative analysis of MC1-positive neurons in the hippocampus, we imaged areas enriched with HA-positive pyramidal neurons in the CA1 and quantified HA and MC1-immunoreactivity in the striatum pyramidale layer using NIH ImageJ software, as described previously [103].

### Immunocytochemistry for Cultured Neurons

Cortical neurons were prepared from E16.5 NMNAT2 WT, HET, or KO embryos and cultured on Poly-D-Lysine coated coverslips. DIV10 neurons were fixed with 4% PFA and 3% Sucrose in PBS and immunostained for synaptic markers Synaptophysin (1/1,000, Millipore, Temecula, CA), and VGlut1 (1/500 (1/1,000, Synaptic Systems, GmbH, Germany), the axonal marker ßIII-Tubulin (1/1,000, Millipore, Temecula, CA) and MAP2 (1/2,000, Millipore, Temecula, CA) as described [104,105]. Cells were imaged using fluorescent Alexa Fluor 555, 488, and 647 conjugated antibodies, with a Leica TCS SPE personal confocal.

### Neuronal Cultures and MTT Assay

Cortical neurons were prepared from the cortices of E16.5 embryos from NMNAT2 wild type, heterozygous, or homozygous [18] using the Worthington Papain Dissociation Kit (LK003153, Worthington Biochemical Corporation, Lakewood, NJ). 50,000 neurons were plated per well in polylysine-D coated 96-well plates (BD Biosciences, San Jose, CA) in Neurobasal media (Gibco, Grand Island, NY). Following plating, half of the media was replenished with B-27 and L-glutamine supplemented fresh Neurobasal media on Days-in-Vitro (DIV2), DIV7 and DIV14 and DIV18. Neurons were treated with prior to treatment with MG132 (10 μM) or DMSO (control) on DIV14 for 12 h. For KCL treatment, neurons were treated at DIV5, 10, 15, or 20 with 30 mM KCl for 24 h. Following either treatment, the MTT assay was performed as described [106]. MTT assay measures cell viability based upon the ability of viable cells to reduce the tetrazolium dye MTT 3-(4,5-dimethylthiazol-2-yl)-2,5-diphenyltetrazolium bromide to its insoluble form formazan [88]. MTT assay was performed on primary cortical neurons grown in 96-well plates, using Vybrant MTT Cell Proliferation Assay Kit, according to manufacturer’s guidelines (Life Technologies). The absorbance values for cells treated with MTT were measured using the 540 nm wavelength, Opsys MR spectrophotometer, and Revelation Quicklink software.

### Lentiviral Transduction in Cortical Neuronal Culture

Wild type NMNAT2, ED, PM, and cATP deletion mutants were cloned into LV provided by the DERC Gene Vector Core at BCM. These LV vectors were concentrated by ultracentrifugation and supplied at roughly 1 x 10^5^ tu/uL. Neurons plated at a concentration of 100K/mL onto Poly-D-Lysine coated coverslips in 24-well plates or 96-well plates for MTT assay, were infected on DIV2 with 1 uL (10^5^) LV particles to transduce almost all the neurons plated. For imaging, coverslips were either fixed at DIV5 or DIV10 with 4%PFA+ 3%Sucrose solution and processed for immunostaining (see below).

### Coimmunoprecipitation

Heads from approximately 5 ml of Transgenic flies carrying P{w[+mW.hs] = GAL4-da.G32}UH1; UAS-HA:Nmnat [1] were homogenized in a dounce homogenizer in lysis buffer containing 25 mM Tris-HCl (pH 7.5), 150 mM NaCl, 5 mM EDTA, 1% NP-40, 1 mM DTT and protease and phosphatase inhibitor (Thermo Scientific, Rockford, IL). The resulting supernatant was collected without centrifugation after 5 min settling on ice and used for coimmunoprecipitation using Sigma Ezview Red Anti-HA affinity gel along with the supplied protocol. Bound proteins were eluted using Sigma HA peptide at 100 ug/ml.

### Mass Spectrometry Analysis

The mass spectrometry analysis was performed in the Mass Spectrometry-Proteomics Core Facility in Baylor College of Medicine. Two microliter aliquots of each sample were quantified using NanoOrange protein quantification kit (Invitrogen). All samples were normalized to one microgram per 100 μL with 50 mM ammonium bicarbonate buffer (pH 7.9). The samples were reduced with 100 mM DTT (BioRad) in a rotary shaker at 800 rpm at room temperature for 30 min. The reduced cysteine residues were further treated with 400 mM iodoacetamide (Sigma) in the dark at room temperature for 30 min in the same shaker. 40 ng of sequencing grade trypsin (Sigma) were added to the samples for digestion. The digests were supplemented with acetonitrile to 10% of total volume. The digestion was allowed to proceed at 37°C for 16 h by mild shaking. Digestion was stopped by the addition of formic acid to 5% final volume and tubes were Speedvac dried. Digests were resuspended in 0.1% formic acid and 5% acetonitrile solution. The concentration of each digest was measured using NanoOrange and 200 ng of each sample was fed into the Eksigent nanoLC system. The Ekisgent nanoLC and the ABCIEX TripleTOF 5,600 mass spectrometer were controlled by Analyst software, version 1.6 (ABCIEX Inc., Framingham, MA).

The Eksigent nanoLC system has a cHiPLC system. The chips contained a trap column (200 μm × 0.5 mm, ChromXP C18-CL, 3 μm, 120Å) that trapped the injected peptides in a flow rate of 3 uL/min in a buffer composed of 50% of 0.1% formic acid and 50% of acetonitrile for 5 min at 23°C. The second section was an analytical column (75 μm × 15 cm, ChromXP C18-CL, 3 μm, 120 Å) with an organic mobile phase gradient set at a 90 min gradient starting from 5 to 35% acetonitrile in 0.1% formic acid at a flow rate of 300 nL/min. The acetonitrile concentration was increased to 80% in 5 min and then held in 80% acetonitrile for an additional 5 min. The final acetonitrile concentration was reduced to 5% within 5 min and then the column was equilibrated for 20 min to prepare for the next sample.

The Eksigent nanoLC was linked via the nanoflex to the ABCIEX TripleTOF 5600 mass spectrometer. The spray tip was the PicoTip emitter (360 μm OD, 20 μm ID, 12 cm long with 10 μm tip opening size) from New Objective. The GS 1 gas was set at 3 units and the curtain gas was set at 24 units. The ionization voltage was set at 2,400 V. The interface heater temperature was set at 150°C. The MS precursor ions were selected from 400 to 1,250 amu. The cycle time was 0.25 s and the forty most abundant ions were allowed to pass to the q2 for product ion production. The MS/MS spectra were acquired at 0.1 second at the m/z range of 100 to 2,000. A beta-galactosidase tryptic digest was used as a calibration standard every sixth run. Raw WIFF files were fed into Protein Pilot version 4.5 (Paragon-based) software (ABCIEX Inc.) for peak generation and database search with <10% false discovery rate. The organism was set as *Drosophila* and the database was the NCBI nonredundant database.

### Ataxin-1 Aggregation Assay

The ataxin-1 aggregation assay was performed as described [87]. Briefly, Cos7 cells were doubly transfected with either pEGFP-hAtaxtn-1[82Q] and pCAG-mCherry, HA-NMNAT2, HA-NMNAT2^W92G^ (ED), HA-NMNAT2^C164S/C165S^ (PM), HA-NMNAT2^ΔNT^, HA-NMNAT2^ΔCT^ or HA-NMNAT2^ΔcATP^ using Lipofectamine (Invitrogen). After 48 h, cells were fixed and immunolabelled to detect HA-tagged NMNATs. Cell nuclei were labeled with DAPI (Molecular Probes, Grand Island, NY). The size of GFP-positive aggregates was measured using Neurolucida software.

To distinguish between soluble and insoluble ataxin, the cells were lysed in RIPA buffer (50 mM Tris (pH 8), 150 mM NaCl, 1 mM EDTA, 1% NP40, 0.5% sodium deoxycholate, 0.1% SDS, 1 mM PMSF and protease inhibitor cocktail (Roche, Branford, CT) and fractionated according to an established protocol [87]. The detergent-soluble fraction was defined as proteins remaining in the supernatant after centrifugation of the cell lysates at 120,000 g for 15 min. After removal of the supernatant, the pellets were washed with RIPA buffer and then suspended in 2% SDS Buffer. One assumption made in this assay was that all cells transfected would have likely chance of incorporating both pEGFP-hAtaxin-1[82Q] and the test chaperone protein.

### Immunofluorescence Staining and Imaging for Cultured Cells

Immunofluorescence staining was performed on cultured cells. Cells were fixed in 4% PFA for 20 min and washed with PBS/0.01% Triton X-100 (PBST) and permeabilized with 0.2% Triton X-100 in PBS at room temperature for 5 min. Next, blocking was performed for 1 h using 5% normal goat serum in PBS. Following this step, the cells were stained for the HA-tag (1:1,000, Cell Signaling) diluted in PBST/1% normal goat serum at 4°C overnight. The next day, cells were washed with PBST, and incubated with goat anti-rabbit or goat anti-mouse 594 secondary antibodies (all at 1/500, Invitrogen) in PBST at room temperature for 2 h. Following this incubation, cells were washed with PBST three times for 10 min each and mounted in Vectashield mounting media with DAPI (Vector Labs, Burlingame, CA) and cover slipped for imaging. Fluorescence microscopy was performed using a Zeiss AxioImager M1 system equipped with epifluorescence filters, a Zeiss monochrome digital camera and AxioVision software. All images were processed in Adobe Photoshop for brightness/contrast, orientation and background correction to better illustrate staining patterns.

### Statistics

Means were compared across groups using one-way analysis of variance (ANOVA) for ≥ three groups or t-tests for two groups for data with normal distribution that met variance homogeneity. Significance was assessed using the Tukey criterion for pairwise mean comparisons under the ANOVA model. Normality was assessed using the Kolmogorov-Smirnov test. When homogeneity of variance was not met under the ANOVA model, a robust ANOVA was carried out. Two-tailed *p*-values <0.05 were considered significant. Values are expressed as mean ± SE. Computations were carried out using Graphpad Prism for Windows, version 6.0 (La Jolla, CA).

## Supporting Information

S1 Data Excel File containing in separate sheets the numerical data for Figs 1E, 2B, 3B, 3D, 3E, 4D–4F, 5B, 5D, 5G, 6A, 6B, 7B, 7D, S1A, S1B, S2, S3, S4A, S4B, S7, S8A, S8B, S9, S11A and S11B. Raw data for Fig 1A, 1B and 1C is derived from RUSH Memory and Religious Orders Study. Sharing these data require a DUA but is “without restriction” upon request (http://www.rush.edu/radc).
(XLSX)

S1 Fig NMNAT2 levels are reduced in AD and nondemented AD neuropathology (NDAN) brains collected by the Oregon Health Center. Both qPCR and western analysis were conducted to determine the levels of *nmnat2* mRNA and protein in postmortem human brains from control and NDAN groups as well as AD patients. NDAN group had normal cognitive function but possessed the same levels of plaque and tangle burden as AD patients (Braak stage of IV through VI and CERAD rating of moderate to frequent; please see S3 Table for individual subjects) [107,108], suggesting that NDAN subjects are resistant to or have extraordinarily delayed the onset of cognitive decline that normally accompanies the progression of fully symptomatic AD. Comparing NDAN to AD cases is a way to shed light on mechanisms that are selectively involved in AD-related cognitive decline. (**A**) The levels of *nmnat1/2* mRNA in postmortem human brains from control (*n* = 5) and NDAN (*n* = 6) groups as well as AD patients (*n* = 6) were measured by quantitative real-time PCR. The levels of mRNA for both genes were normalized to GAPDH and 18s mRNA loading controls. We found a significant reduction in *nmnat2* mRNA levels in NDAN and an even greater decline in AD brains. There was no change of *nmnat1* abundance in NDAN or AD groups. (**B**) Quantification of NMNAT2 protein levels in soluble fractions from Control, NDAN and AD brains reveals a reduction in NMNAT2 levels in NDAN and AD, consistent with the decreased mRNA level. The majority of proteins extracted from these human brain samples is present in the soluble fraction, which extracts both soluble and membrane bound proteins. Comparatively, a very small fraction of proteins, comprised mainly of aggregated pathological species and proteins bound to such species appear in the insoluble fraction (approximately 1/100 of the total amount present in the soluble fraction) [109]. Error bars represent SEM. The differential reduction of NMNAT2 in NDAN and AD suggests that the reduction of NMNAT2 below a threshold contributes to cognitive decline in AD. *p<0.05, **p < 0.01, ***p < 0.001 when compared to control. Raw data for S1A and S1B Fig can be found in S1 Data.
(TIF)

S2 Fig Mammalian NMNATs act as chaperones to refold and prevent heat-denatured luciferase aggregation. Mammalian NMNATs act as chaperones to refold and prevent heat-denatured luciferase aggregation. Cos7 cells were transfected with cytoplasmic luciferase together with either mCherry, human NMNAT1, 2 or 3 or HSP70. In this assay, heat denaturation along with the protein-synthesis inhibitor cycloheximide, renders the endogenous chaperone machinery incapable of preventing luciferase aggregation and recovery post stress, allowing for the chaperone activity of an introduced test protein to be directly measured. The cells expressing luciferase and various proteins were briefly heated at 45°C (heat shock), returned to 37°C and the luciferase activity was compared before and after heat shock to assess the ability of test proteins to protect luciferase from heat shock-induced denaturation (holdase activity) and to promote proper refolding of heat-denatured luciferase during recovery (foldase activity). Summary shows that the presence of HSP70 or NMNATs 1, 2, and 3 reduced the aggregation of luciferase post heat shock and promoted refolding of the aggregated luciferase during the recovery phase (3 independent experiments with triplicate in each experiment). Blue bars show baseline luciferase activity. Red bars show luciferase activity immediately after 15 min 42°C heat shock while blue bars show luciferase activity after 3 hours of post-heat shock recovery. *p < 0.05 when compared to mCherry HS, #*p* < 0.05 when compared to mCherry Recovery. Raw data can be found in S1 Data.
(TIF)

S3 Fig NMNATs act as molecular chaperones. (**A**) Example data from an in vitro chaperone activity assay. In this assay, recombinant CS is induced to aggregate with thermal stress, and the aggregation process quantified by Rayleigh scattering of the CS homoaggregates versus time. CS was incubated with Lysozyme (negative control), HSP70 (positive control), or human NMNAT1-3 for 30 min. Aggregation of CS was induced at 43°C and monitored as absorbance at 350 nm over time. Decreased absorbance corresponds to less aggregation, which could result from a protein binding to CS and stabilizing it to protect it from aggregation. Incubation of CS with increasing amounts of purified recombinant hNMNAT1, 2, 3, or HSP70 resulted in a concomitant decrease in substrate aggregation after thermal denaturation in a dose-dependent manner. At the same time, lysozyme did not have any effect on CS aggregation. This assay reveals the holdase activity of HSP70 and NMNAT1-3, by which they can bind to aggregation-prone substrates and keep them in a stable phase to prevent aggregation. (**B**) Summary showing that CS aggregation was reduced in a dose-dependent manner by recombinant HSP70, NMNAT1, 2, or 3, but not lysozyme. The potency of human NMNAT1–3 to decrease substrate denaturation was similar to that of HSP70, a well-studied chaperone. Raw data can be found in S1 Data.
(TIF)

S4 Fig Enzymatic and stability properties of NMNAT2 variants. (**A**) Enzyme activity of WT and various NMNAT2 mutants, in Cos7 cells, as measured by NAD and NADH levels (*n* = 3 experiments). ED and ΔNT show similar NAD/NADH levels as the mCherry control, proving that these constructs are indeed enzymatically inactive. PM is more stable and correspondingly shows more enzymatic activity. ΔCT and ΔcATP mutants exhibit similar enzymatic functions as WT NMNAT2. (B) NAD levels in hippocampus after 1 mo postinjection of AAV overexpressing GFP or NMNAT2-WT, -ED, or -ΔcATP. Clearly, overexpressing -ED does not increase NAD levels as seen with -WT or -ΔcATP. (C) The stability of WT and mutant NMNAT2 was measured in Cos7 cells treated with 10 μM cycloheximide for 4 h (*n* = 3 independent experiments). Among NMNATs, NMNAT2 is the most labile, with a half-life less than 2 h [15,16]. Deleting the central domain or mutating the palmitoylation sites (C164 and 165) in this domain significantly increases NMNAT2’s stability [17]. The N- and C-terminal domains of NMNAT2 are highly conserved with those of NMNAT1 and 3, while its central domain is unique [68]. Deleting the N or C-terminal domain (ΔNT and ΔCT) had no impact on NMNAT2 stability. The half-lives of NMNAT- ED mutant, the C-terminal ATP site deletion (ΔcATP) mutant, and NMNAT2-WT were all similar. * indicate significant differences from mCherry-(B)/GFP (C)-NAD, and # indicate significant differences from mCherry-NADH, respectively. *p < 0.05, **p < 0.001, and ****^/####^ *p* < 0.0001. Raw data for S4A and S4B Fig can be found in S1 Data.
(TIF)

S5 Fig NMNAT2 appears in the insoluble fraction in rTg4510 cortex, complexing with Tau and HSP90. (A) In the rTg4510 tauopathy model, where FTD-linked mutant human Tau is overexpressed in the forebrain from 1 mo of age. Neurodegeneration phenotypes in this mouse line become evident from 2.5 mo of age [110]. In this model, tau hyperphosphorylation is observed from 2.5 mo of age [111] and increases with age. NMNAT2 shifts solubility in 6-mo-old rTg4510 brains and a substantial fraction of NMNAT2 became insoluble, colocalizing with tau oligomers and HSP90 in the insoluble fraction, while in control brains almost no NMNAT2 was found in the insoluble fraction. A similar change in HSP90 solubility was observed in human AD cortices, but not in control. (B) Previously, we reported that NMNAT2 levels decline in this model as early as 1 mo of age, preceding the onset of neurodegeneration or behavioral impairments. When we investigated when NMNAT2 protein shifts solubility NMNAT2 in this model, we observed that this occurs simultaneously as Tau becomes insoluble. rTg4510 cortices, where P301L-mutant human Tau was overexpressed with a neuronal CAMKII promoter, were harvested at age 1 and 3 mo. In young mice, where hTau insolubility had not occurred, NMNAT2 was only detected in the soluble fraction. However, at 3 mo of age, NMNAT2 became insoluble, with hTau insoluble oligomers detected by AT8 Ab. AT8 Ab detects hTau phosphorylated at Ser202 and Thr 205 [112]. At this same time point, NMNAT2 levels were reduced in the soluble fraction. Total tubulin was used as a loading control.
(TIF)

S6 Fig NMNAT2’s C-terminal domain is required to complex with HSP90. (**A**) In this stable cell line, 2N4R tau (hTau40) is overexpressed when doxycycline (1 μg/mL) is added to the media [77]. Over time, hyperphosphorylated Tau forms and aggregates. 24 h after inducing hTau expression, HA-NMNAT2 was overexpressed in these cells. 48 h post transfection, the cells were lysed and HA-tagged proteins immunoprecipitated from the soluble fraction. Immunoprecipitation of HA-tagged NMNAT2 from soluble lysates of hTau40 stable cell line reveals efficient pull down of all NMNAT2 variants. HSP90 was immunoprecipitated with all but the C-terminal NMNAT2 deletion mutant. (**B**) The interaction between HSP90 and NMNAT2 in cells overexpressing Ataxin1-82Q-GFP also requires the C-terminal of NMNAT2. Experiment repeated three independent times.
(TIF)

S7 Fig Inhibiting or reducing HSP90 function increases luciferase activity after heat shock and following the recovery phase. During basal conditions (nonstressed), HSP90 binds to HSF1 and p23-immunophilins [83]. This complex prevents HSF1 from forming homo trimers and activating the transcription of the heat shock response genes (Fig 4C) [83]. Hence, reduction or inhibition of HSP90 by treating the cells with siRNA or 17-AAG promotes the heat shock response by increasing free HSF1 and HSP transcription. Here, we found that reducing HSP90 activity lessened luciferase aggregation and promoted refolding post heat shock in mCherry-expressing cells. Such an effect is likely to arise from overactivation of HSF1 upon inhibition of HSP90. Indeed, coapplication of KRI, which blocks HSF1 recruitment to Heat Shock Elements [86], reversed the effects of blocking HSP90 on luciferase refolding. Bar graph showing the summary of luciferase assay in the mCherry-expressing cells with various treatments. Experiments were repeated four times in triplicate. * indicates *p* < 0.05 when compared to mCherry HS or Recovery respectively. Raw data can be found in S1 Data.
(TIF)

S8 Fig NMNAT2’s interaction with HSP90 activates its ATPase activity. **(A)** ATPase activity of NMNAT2 was measured after 5 min of incubation with reactants in the presence of 500 uM ATP. NMNATs catalyze the condensation of ATP with NMN to form NAD and pyrophosphate (PPi). However, this assay only detects the release of inorganic phosphate. To test the enzyme efficacy of the NMNATs, we added pyrophosphatase that will release inorganic phosphate from pyrophosphate. In the presence of aggregated CS, NMNAT2 lacks ATPase activity. However, addition of pyrophosphatase yields inorganic phosphate in this reaction when NMN is present (red), showing that our protein is enzymatically active. (B) When we add HSP90 with NMNAT2 and aggregated CS, we activate NMNAT2’s ATPase activity, which can be estimated by subtracting the Pi released in this ATPase reaction from that containing only HSP90 (last blue bar on right). Experiments were repeated three times. **p < 0.001, ***p < 0.0001 respectively in three independent experiments. Raw data for S8A and S8B Fig can be found in S1 Data.
(TIF)

S9 Fig NMNAT2:HSP90 complexing hydrolyzes ATP at the C-terminus ATP site. Mutating NMNAT2 to the ΔcATP mutant abolishes the ATPase activity of NMNAT2 in the presence of HSP90. Note, the blue bars with ΔcATP+HSP90 are similar to those with just HSP90 in the reaction. This suggests that ATP bound to the C-terminal ATP site of NMNAT2 in the presence of aggregated substrates is only hydrolyzed when NMNAT2 complexes with HSP90. Experiments were repeated three times. *p < 0.05, ***p < 0.0001 and ns = not significant, respectively, in three independent experiments. Raw data can be found in S1 Data.
(TIF)

S10 Fig NMNAT2 KO neurons survive in culture, showing age-dependent degeneration. MTT assay measures neuronal viability indirectly by measuring mitochondrial activity. Assay was performed in three independent experiments for neurons plated at 50,000 cells per well in 96-well plates at the designated time points post plating. (**A**) Summary of MTT reduction in NMNAT2 WT, HET and KO cortical neurons at different time-points post plating (*n* = 3 independent experiments). (**B**) Summary of MTT reduction in NMNAT2 WT, HET and KO cortical neurons after KCl treatment (*n* = 3 independent experiments). Experiments were repeated three times. ns = not significant, *p < 0.05, **p < 0.001
(TIF)

S11 Fig Treatment of NMNAT2 KO neurons with KCl or MG132 causes dendritic blebbing. (A) Example images show that immunostaining with dendritic marker MAP2 (red) allowed us to visualize dying neurons with blebbing dendrites [113,114]. DAPI (blue)-stained nuclei. WT or NMNAT2 KO DIV14 neurons were treated with 30 mM KCl or 10 uM MG132 for 24 h. (B) Sample images of MAP2 immunostaining for NMNAT2 KO neurons overexpressing GFP or NMNAT2 wildtype or NMNAT2 variants. These neurons were treated with either KCl or MG132 as indicated. Raw data for S11A and S11B Fig can be found in S1 Data.
(TIF)

S12 Fig Levels of synaptic vesicle proteins are reduced in NMNAT2 KO neurons. Example images of immunostaining of cultured NMNAT2 WT, HET and KO neurons at DIV10 with an axonal marker (BIII-Tubulin) and synaptic vesicle markers VGlut1 (A) or Synaptophysin (B). White box highlights region magnified in the right panels to show synaptic vesicle trafficking along axonal segments. Experiments were repeated three times.
(TIF)

S13 Fig NMNAT2 only interacts with HSP90 in proteotoxic stress conditions. Cos7 cells were transfected with HA-NMNAT2, and after 48 h post transfection, they were treated with either DMSO or MG132 for 12 h. Lysates were used to immunoprecipitate with HSP90 or HA-NMNAT2 antibodies. IgG was used as negative control. In: Input; IP: Immunoprecipitate.
(TIF)

S14 Fig A simplified model of NMNAT2’s chaperoning activity. A simplified model of NMNAT2’s chaperoning activity. Upon proteotoxic stress, NMNAT2 binds to aggregation-prone proteins and maintains them in a nascent form while subsequent interactions with HSP90 promote refolding of aggregated substrates.
(TIF)

S1 Table Characteristics of the ROS/MAP clinicopathologic cohort. (DOCX)

S2 Table Association of *nmnat2* transcript levels with cognitive decline and AD pathologic burden. Linear regression models examining transcript levels in relation to the global cognition or global AD pathology measures. All analyses were adjusted for age of death, sex, postmortem interval, and RIN score. In a secondary analysis (*), the regression model was additionally adjusted for time from last clinical evaluation to death.
(DOCX)

S3 Table Characteristics of Control, AD, and NDAN cohort used for validation and biochemistry. Braak: 6-many tangles, 0-no tangles; Plaque: 1-high plaque load, 4-no plaque.
(DOCX)

S4 Table Proteins identified by mass spectrometry as potentially physically interacting with dNMNAT. % coverage gives the number of amino acids in sequenced peptides/the total number of amino acids in the protein. Accession gives the accession number from the NCBI nonredundant database with the organism set to *Drosophila*. *Name is the current accepted protein name as listed in Flybase.org.
(DOCX)

S5 Table Disease-specific changes in *nmnat2* transcripts mined from NextBio, along with statistical significance and specific references of the published studies that have deposited the specific microarray data on publicly available repositories. (DOCX)

## Abbreviations

AD: Alzheimer disease
ADC: Layton Aging and AD Center
CDR: clinical dementia rating
CHIP: C-terminus of HSC70 Interacting Protein
CNS: central nervous system
CS: citrate synthase
CSPα: cysteine string protein α
DIV: days-in-vitro
DTT: dithiothreitol
*d*NMNAT: *Drosophila* NMNAT
ED: enzyme-dead
EF-1: elongation factor 1
FPKM: Fragment Per Kilobase Per Million
FTDP-17: Frontotemporal Dementia and Parkinsonism-17
Hsc70: heat shock cognate 70
HSF1: Heat Shock Factor 1
KRI: KRIBB-11
LV: lentiviral vector
MCI: mild cognitive impairment
MESG: 2-amino-6-mercapto-7-methylpurine riboside
NAD: nicotinamide adenine dinucleotide
NDAN: nondemented AD neuropathology
NMNAT: nicotinamide mononucleotide adenylyl transferase
NR1: N-methyl-D-aspartate receptor 1
p-hTau: hyperphosphorylated human Tau
PFA: paraformaldehyde
PNP: purine nucleoside phosphorylase
rAAV: recombinant adenoassociated virus
RIN: RNA integrity number
SCA1: Spinocerebellar Ataxia type 1
sd: standard deviation
