# Supplementary figures and images for: NMNAT2:HSP90 Complex Mediates Proteostasis in Proteinopathies
Source: PLoS Biol. 2016 Jun 2;14(6):e1002472. doi: 10.1371/journal.pbio.1002472 (PMC4890852; doi:10.1371/journal.pbio.1002472)

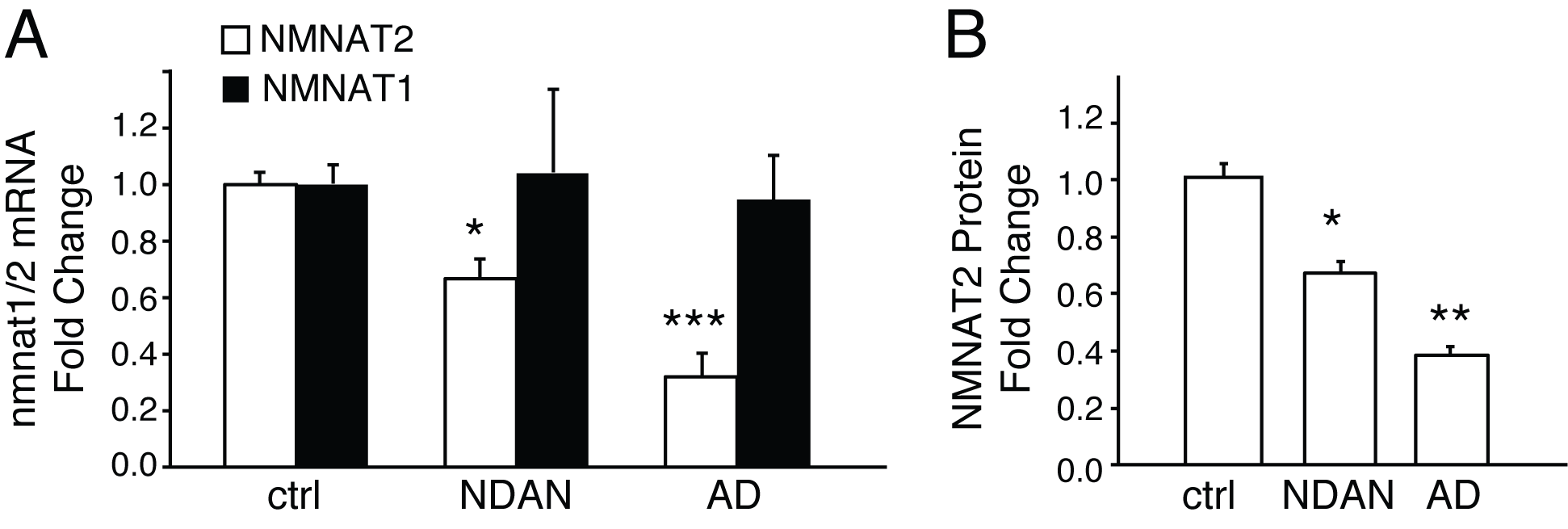

Supplement: S1 Fig — Both qPCR and western analysis were conducted to determine the levels of nmnat2 mRNA and protein in postmortem human brains from control and NDAN groups as well as AD patients. NDAN group had normal cognitive function but possessed the same levels of plaque and tangle burden as AD patients (Braak stage of IV through VI and CERAD rating of moderate to frequent; please see S3 Table for individual subjects) [107,108], suggesting that NDAN subjects are resistant to or have extraordinarily delayed the onset of cognitive decline that normally accompanies the progression of fully symptomatic AD. Comparing NDAN to AD cases is a way to shed light on mechanisms that are selectively involved in AD-related cognitive decline. (A) The levels of nmnat1/2 mRNA in postmortem human brains from control (n = 5) and NDAN (n = 6) groups as well as AD patients (n = 6) were measured by quantitative real-time PCR. The levels of mRNA for both genes were normalized to GAPDH and 18s mRNA loading controls. We found a significant reduction in nmnat2 mRNA levels in NDAN and an even greater decline in AD brains. There was no change of nmnat1 abundance in NDAN or AD groups. (B) Quantification of NMNAT2 protein levels in soluble fractions from Control, NDAN and AD brains reveals a reduction in NMNAT2 levels in NDAN and AD, consistent with the decreased mRNA level. The majority of proteins extracted from these human brain samples is present in the soluble fraction, which extracts both soluble and membrane bound proteins. Comparatively, a very small fraction of proteins, comprised mainly of aggregated pathological species and proteins bound to such species appear in the insoluble fraction (approximately 1/100 of the total amount present in the soluble fraction) [109]. Error bars represent SEM. The differential reduction of NMNAT2 in NDAN and AD suggests that the reduction of NMNAT2 below a threshold contributes to cognitive decline in AD. *p<0.05, **p < 0.01, ***p < 0.001 when compared to control. Raw [file pbio.1002472.s002.tif]

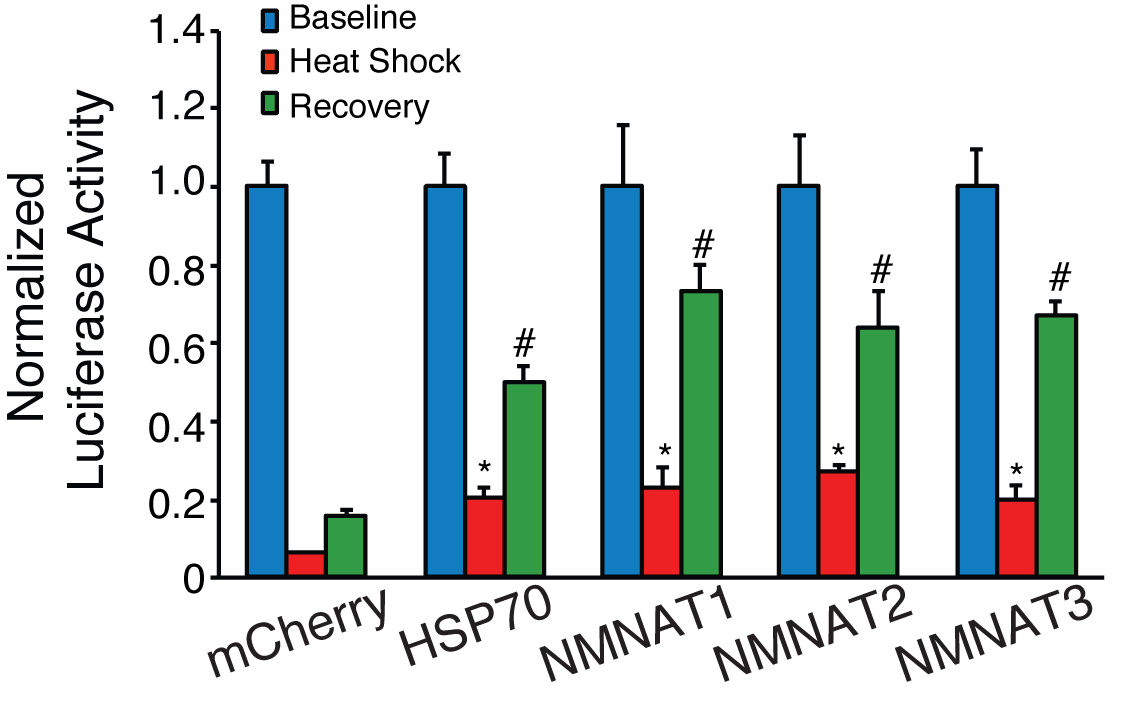

Supplement: S2 Fig — Mammalian NMNATs act as chaperones to refold and prevent heat-denatured luciferase aggregation. Cos7 cells were transfected with cytoplasmic luciferase together with either mCherry, human NMNAT1, 2 or 3 or HSP70. In this assay, heat denaturation along with the protein-synthesis inhibitor cycloheximide, renders the endogenous chaperone machinery incapable of preventing luciferase aggregation and recovery post stress, allowing for the chaperone activity of an introduced test protein to be directly measured. The cells expressing luciferase and various proteins were briefly heated at 45°C (heat shock), returned to 37°C and the luciferase activity was compared before and after heat shock to assess the ability of test proteins to protect luciferase from heat shock-induced denaturation (holdase activity) and to promote proper refolding of heat-denatured luciferase during recovery (foldase activity). Summary shows that the presence of HSP70 or NMNATs 1, 2, and 3 reduced the aggregation of luciferase post heat shock and promoted refolding of the aggregated luciferase during the recovery phase (3 independent experiments with triplicate in each experiment). Blue bars show baseline luciferase activity. Red bars show luciferase activity immediately after 15 min 42°C heat shock while blue bars show luciferase activity after 3 hours of post-heat shock recovery. *p < 0.05 when compared to mCherry HS, #p < 0.05 when compared to mCherry Recovery. Raw data can be found in S1 Data. (TIF) [file pbio.1002472.s003.tif]

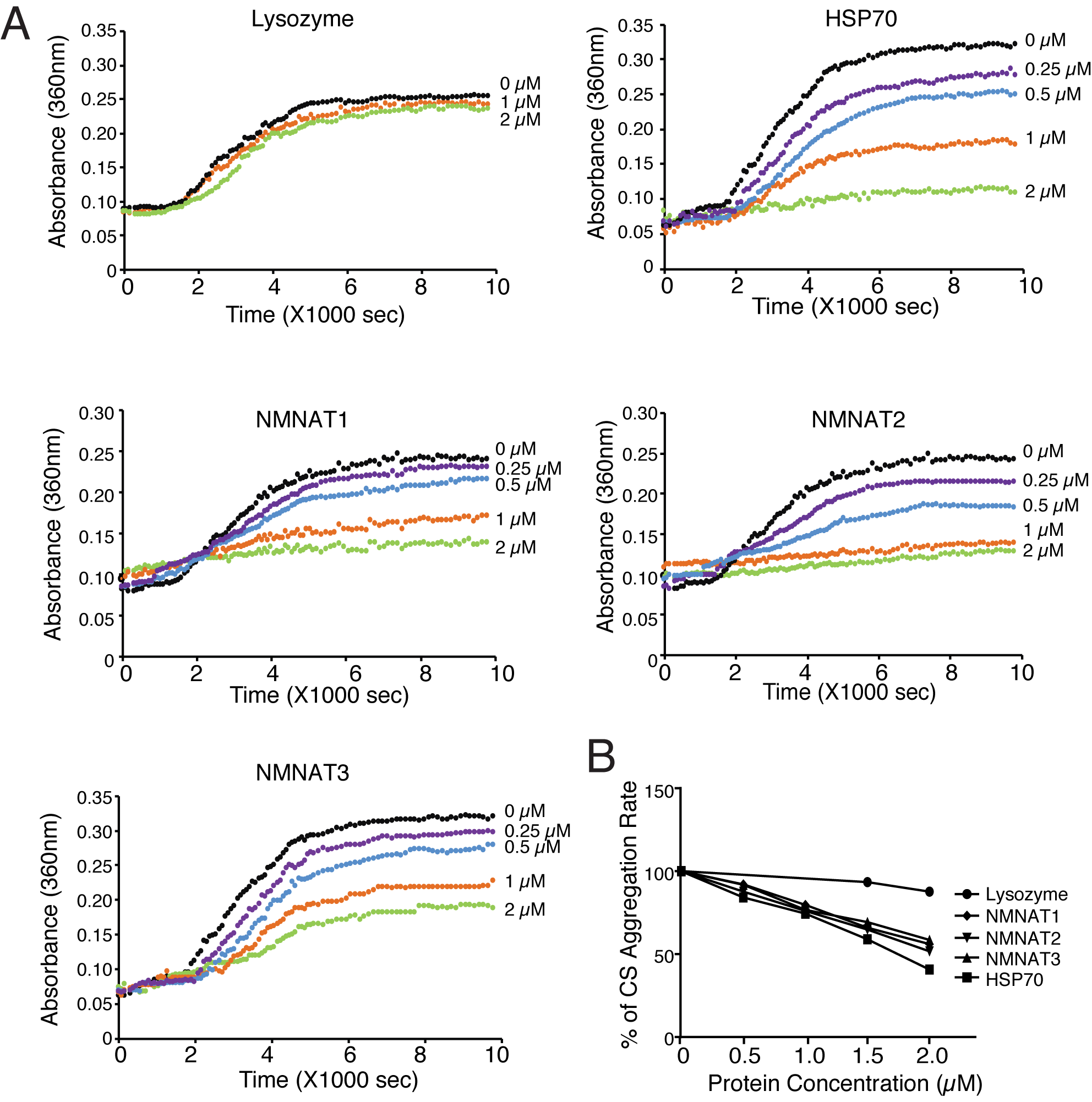

Supplement: S3 Fig — (A) Example data from an in vitro chaperone activity assay. In this assay, recombinant CS is induced to aggregate with thermal stress, and the aggregation process quantified by Rayleigh scattering of the CS homoaggregates versus time. CS was incubated with Lysozyme (negative control), HSP70 (positive control), or human NMNAT1-3 for 30 min. Aggregation of CS was induced at 43°C and monitored as absorbance at 350 nm over time. Decreased absorbance corresponds to less aggregation, which could result from a protein binding to CS and stabilizing it to protect it from aggregation. Incubation of CS with increasing amounts of purified recombinant hNMNAT1, 2, 3, or HSP70 resulted in a concomitant decrease in substrate aggregation after thermal denaturation in a dose-dependent manner. At the same time, lysozyme did not have any effect on CS aggregation. This assay reveals the holdase activity of HSP70 and NMNAT1-3, by which they can bind to aggregation-prone substrates and keep them in a stable phase to prevent aggregation. (B) Summary showing that CS aggregation was reduced in a dose-dependent manner by recombinant HSP70, NMNAT1, 2, or 3, but not lysozyme. The potency of human NMNAT1–3 to decrease substrate denaturation was similar to that of HSP70, a well-studied chaperone. Raw data can be found in S1 Data. (TIF) [file pbio.1002472.s004.tif]

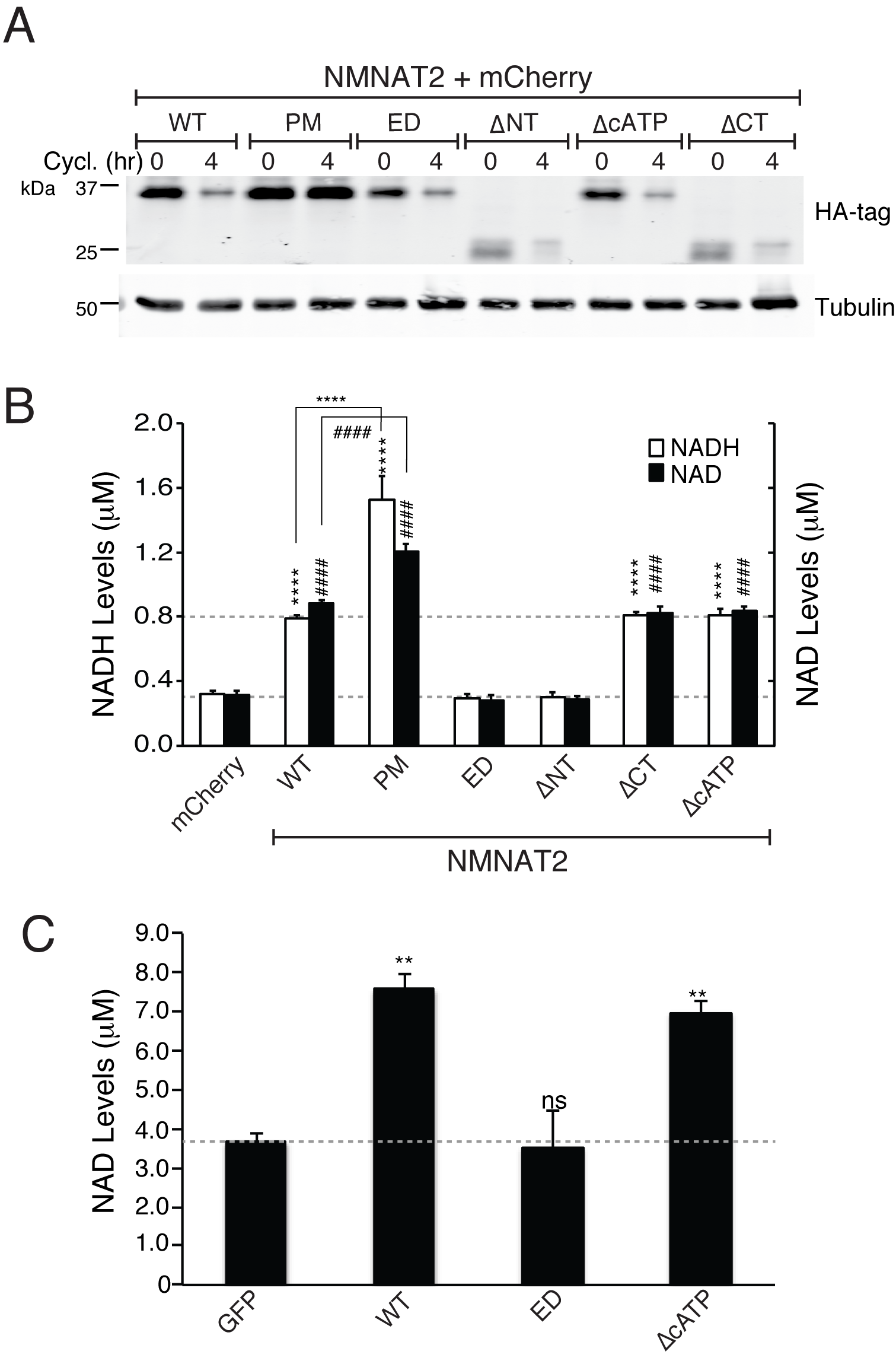

Supplement: S4 Fig — (A) Enzyme activity of WT and various NMNAT2 mutants, in Cos7 cells, as measured by NAD and NADH levels (n = 3 experiments). ED and ΔNT show similar NAD/NADH levels as the mCherry control, proving that these constructs are indeed enzymatically inactive. PM is more stable and correspondingly shows more enzymatic activity. ΔCT and ΔcATP mutants exhibit similar enzymatic functions as WT NMNAT2. (B) NAD levels in hippocampus after 1 mo postinjection of AAV overexpressing GFP or NMNAT2-WT, -ED, or -ΔcATP. Clearly, overexpressing -ED does not increase NAD levels as seen with -WT or -ΔcATP. (C) The stability of WT and mutant NMNAT2 was measured in Cos7 cells treated with 10 μM cycloheximide for 4 h (n = 3 independent experiments). Among NMNATs, NMNAT2 is the most labile, with a half-life less than 2 h [15,16]. Deleting the central domain or mutating the palmitoylation sites (C164 and 165) in this domain significantly increases NMNAT2’s stability [17]. The N- and C-terminal domains of NMNAT2 are highly conserved with those of NMNAT1 and 3, while its central domain is unique [68]. Deleting the N or C-terminal domain (ΔNT and ΔCT) had no impact on NMNAT2 stability. The half-lives of NMNAT- ED mutant, the C-terminal ATP site deletion (ΔcATP) mutant, and NMNAT2-WT were all similar. * indicate significant differences from mCherry-(B)/GFP (C)-NAD, and # indicate significant differences from mCherry-NADH, respectively. *p < 0.05, **p < 0.001, and ****/#### p < 0.0001. Raw data for S4A and S4B Fig can be found in S1 Data. (TIF) [file pbio.1002472.s005.tif]

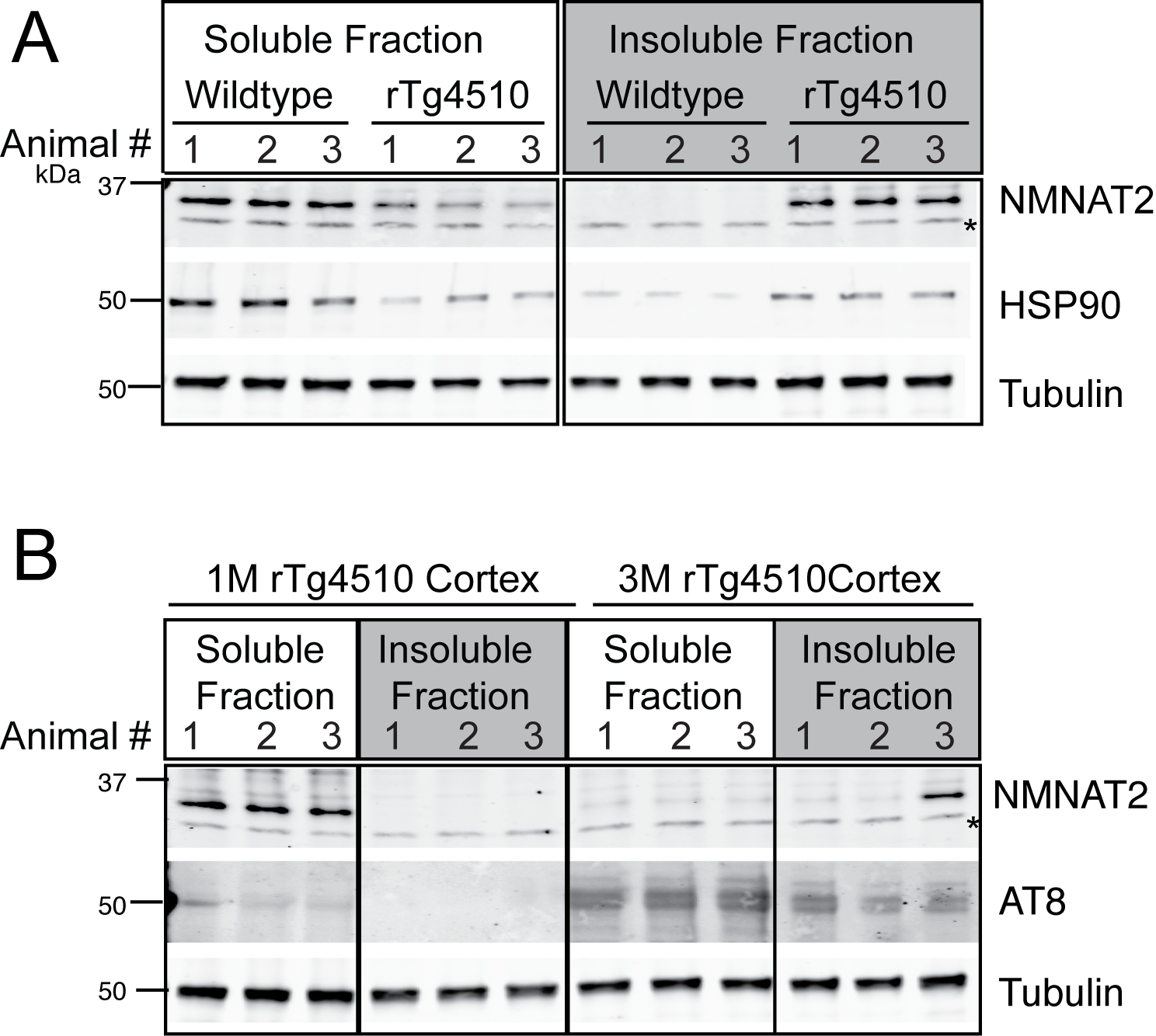

Supplement: S5 Fig — (A) In the rTg4510 tauopathy model, where FTD-linked mutant human Tau is overexpressed in the forebrain from 1 mo of age. Neurodegeneration phenotypes in this mouse line become evident from 2.5 mo of age [110]. In this model, tau hyperphosphorylation is observed from 2.5 mo of age [111] and increases with age. NMNAT2 shifts solubility in 6-mo-old rTg4510 brains and a substantial fraction of NMNAT2 became insoluble, colocalizing with tau oligomers and HSP90 in the insoluble fraction, while in control brains almost no NMNAT2 was found in the insoluble fraction. A similar change in HSP90 solubility was observed in human AD cortices, but not in control. (B) Previously, we reported that NMNAT2 levels decline in this model as early as 1 mo of age, preceding the onset of neurodegeneration or behavioral impairments. When we investigated when NMNAT2 protein shifts solubility NMNAT2 in this model, we observed that this occurs simultaneously as Tau becomes insoluble. rTg4510 cortices, where P301L-mutant human Tau was overexpressed with a neuronal CAMKII promoter, were harvested at age 1 and 3 mo. In young mice, where hTau insolubility had not occurred, NMNAT2 was only detected in the soluble fraction. However, at 3 mo of age, NMNAT2 became insoluble, with hTau insoluble oligomers detected by AT8 Ab. AT8 Ab detects hTau phosphorylated at Ser202 and Thr 205 [112]. At this same time point, NMNAT2 levels were reduced in the soluble fraction. Total tubulin was used as a loading control. (TIF) [file pbio.1002472.s006.tif]

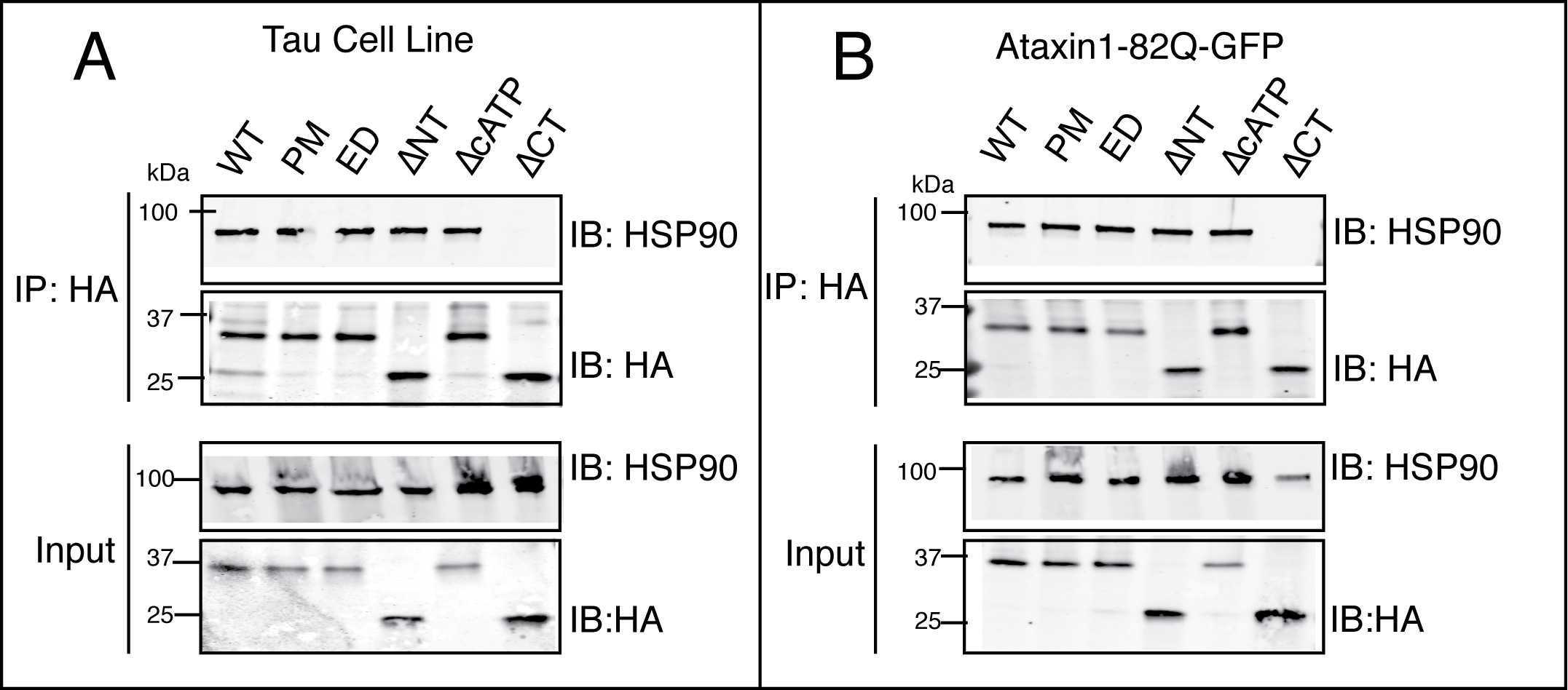

Supplement: S6 Fig — (A) In this stable cell line, 2N4R tau (hTau40) is overexpressed when doxycycline (1 μg/mL) is added to the media [77]. Over time, hyperphosphorylated Tau forms and aggregates. 24 h after inducing hTau expression, HA-NMNAT2 was overexpressed in these cells. 48 h post transfection, the cells were lysed and HA-tagged proteins immunoprecipitated from the soluble fraction. Immunoprecipitation of HA-tagged NMNAT2 from soluble lysates of hTau40 stable cell line reveals efficient pull down of all NMNAT2 variants. HSP90 was immunoprecipitated with all but the C-terminal NMNAT2 deletion mutant. (B) The interaction between HSP90 and NMNAT2 in cells overexpressing Ataxin1-82Q-GFP also requires the C-terminal of NMNAT2. Experiment repeated three independent times. (TIF) [file pbio.1002472.s007.tif]

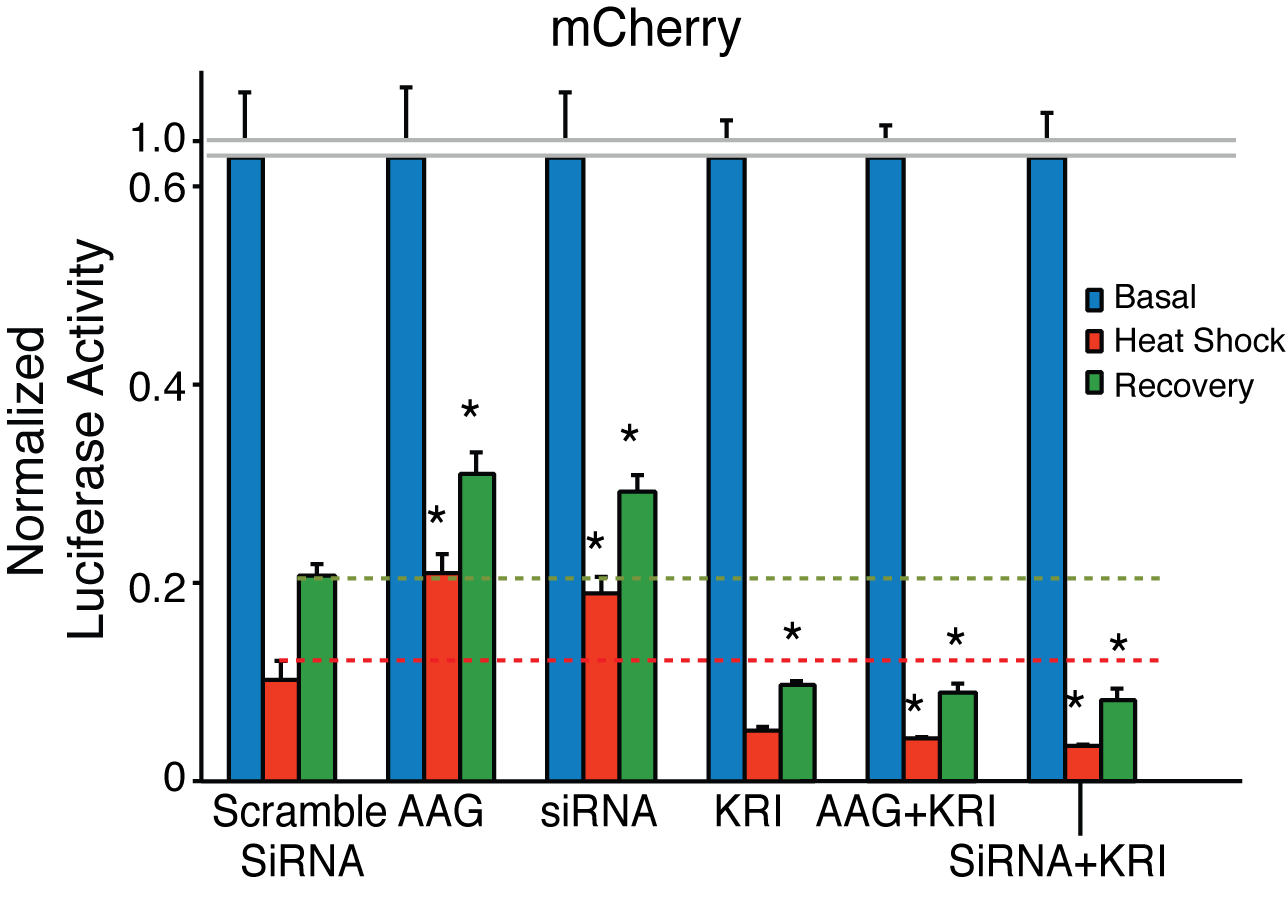

Supplement: S7 Fig — During basal conditions (nonstressed), HSP90 binds to HSF1 and p23-immunophilins [83]. This complex prevents HSF1 from forming homo trimers and activating the transcription of the heat shock response genes (Fig 4C) [83]. Hence, reduction or inhibition of HSP90 by treating the cells with siRNA or 17-AAG promotes the heat shock response by increasing free HSF1 and HSP transcription. Here, we found that reducing HSP90 activity lessened luciferase aggregation and promoted refolding post heat shock in mCherry-expressing cells. Such an effect is likely to arise from overactivation of HSF1 upon inhibition of HSP90. Indeed, coapplication of KRI, which blocks HSF1 recruitment to Heat Shock Elements [86], reversed the effects of blocking HSP90 on luciferase refolding. Bar graph showing the summary of luciferase assay in the mCherry-expressing cells with various treatments. Experiments were repeated four times in triplicate. * indicates p < 0.05 when compared to mCherry HS or Recovery respectively. Raw data can be found in S1 Data. (TIF) [file pbio.1002472.s008.tif]

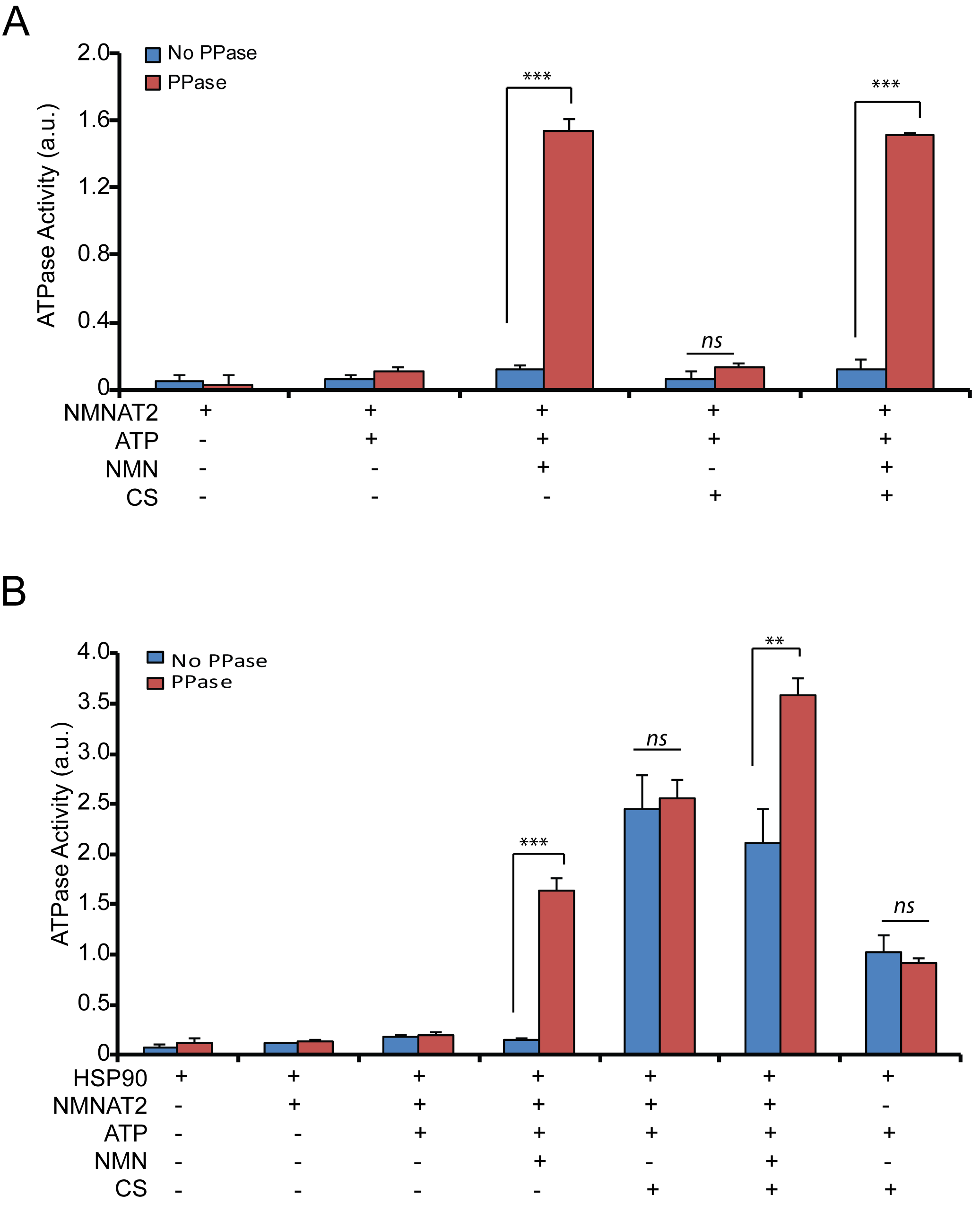

Supplement: S8 Fig — (A) ATPase activity of NMNAT2 was measured after 5 min of incubation with reactants in the presence of 500 uM ATP. NMNATs catalyze the condensation of ATP with NMN to form NAD and pyrophosphate (PPi). However, this assay only detects the release of inorganic phosphate. To test the enzyme efficacy of the NMNATs, we added pyrophosphatase that will release inorganic phosphate from pyrophosphate. In the presence of aggregated CS, NMNAT2 lacks ATPase activity. However, addition of pyrophosphatase yields inorganic phosphate in this reaction when NMN is present (red), showing that our protein is enzymatically active. (B) When we add HSP90 with NMNAT2 and aggregated CS, we activate NMNAT2’s ATPase activity, which can be estimated by subtracting the Pi released in this ATPase reaction from that containing only HSP90 (last blue bar on right). Experiments were repeated three times. **p < 0.001, ***p < 0.0001 respectively in three independent experiments. Raw data for S8A and S8B Fig can be found in S1 Data. (TIF) [file pbio.1002472.s009.tif]

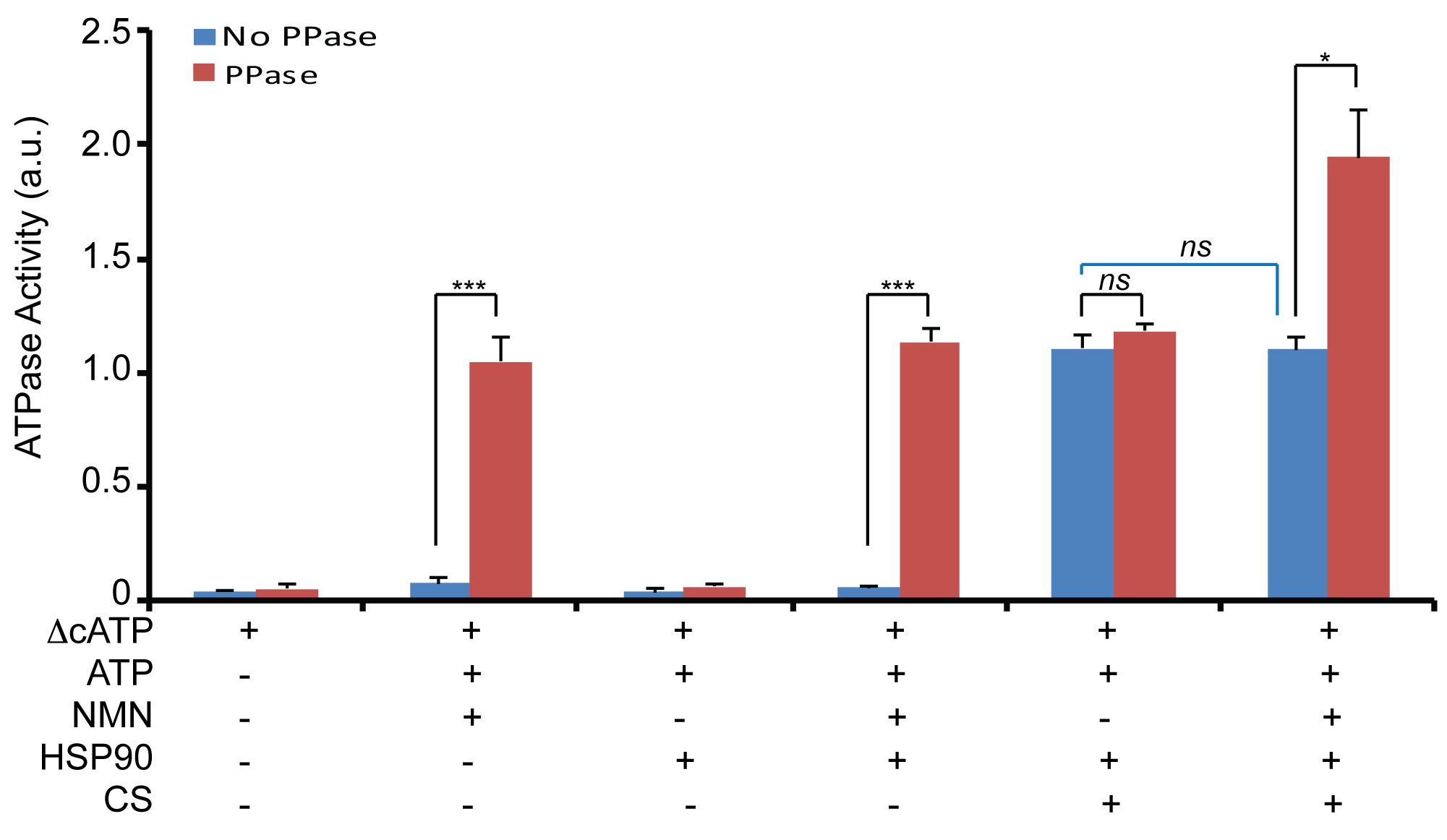

Supplement: S9 Fig — Mutating NMNAT2 to the ΔcATP mutant abolishes the ATPase activity of NMNAT2 in the presence of HSP90. Note, the blue bars with ΔcATP+HSP90 are similar to those with just HSP90 in the reaction. This suggests that ATP bound to the C-terminal ATP site of NMNAT2 in the presence of aggregated substrates is only hydrolyzed when NMNAT2 complexes with HSP90. Experiments were repeated three times. *p < 0.05, ***p < 0.0001 and ns = not significant, respectively, in three independent experiments. Raw data can be found in S1 Data. (TIF) [file pbio.1002472.s010.tif]

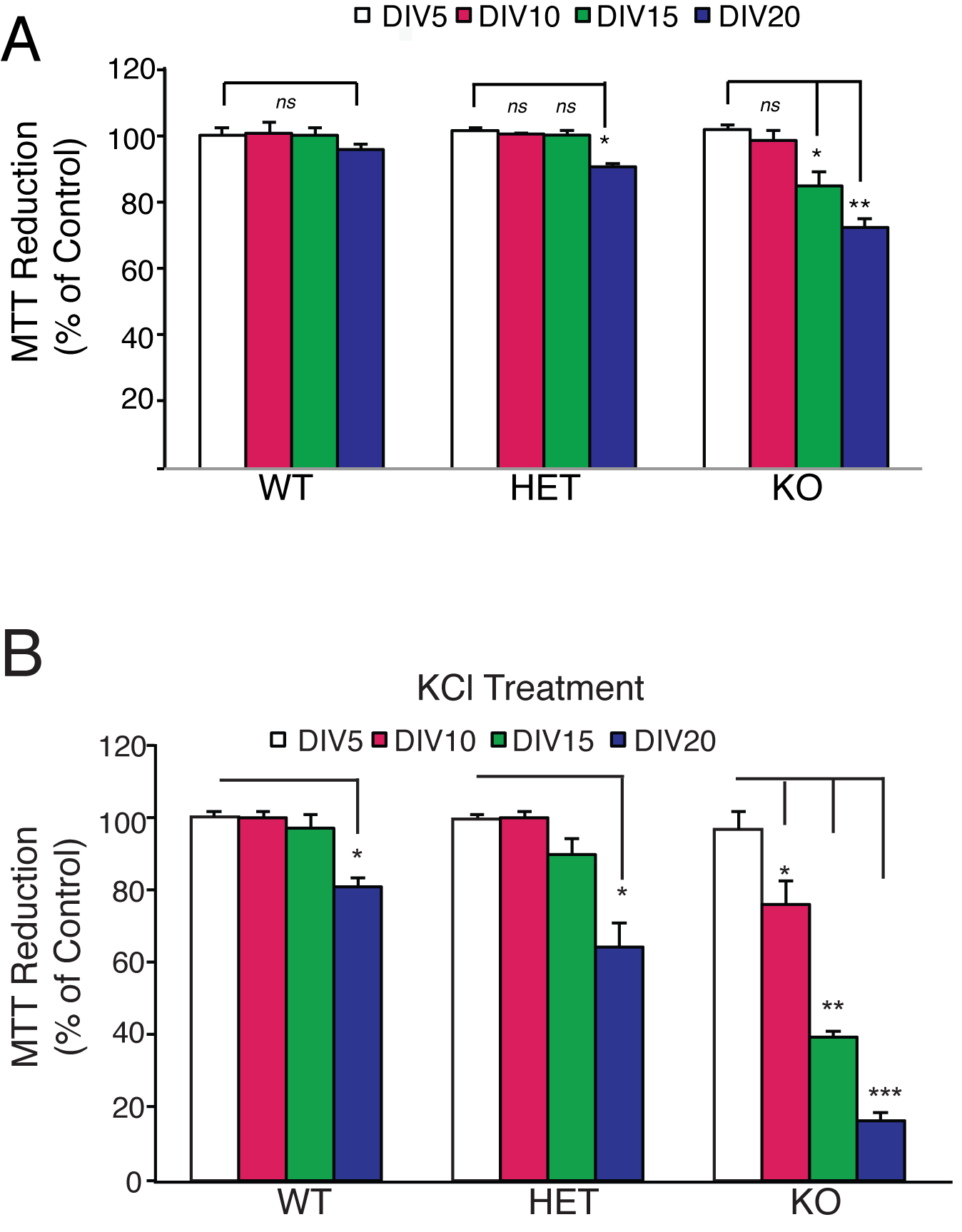

Supplement: S10 Fig — MTT assay measures neuronal viability indirectly by measuring mitochondrial activity. Assay was performed in three independent experiments for neurons plated at 50,000 cells per well in 96-well plates at the designated time points post plating. (A) Summary of MTT reduction in NMNAT2 WT, HET and KO cortical neurons at different time-points post plating (n = 3 independent experiments). (B) Summary of MTT reduction in NMNAT2 WT, HET and KO cortical neurons after KCl treatment (n = 3 independent experiments). Experiments were repeated three times. ns = not significant, *p < 0.05, **p < 0.001 (TIF) [file pbio.1002472.s011.tif]

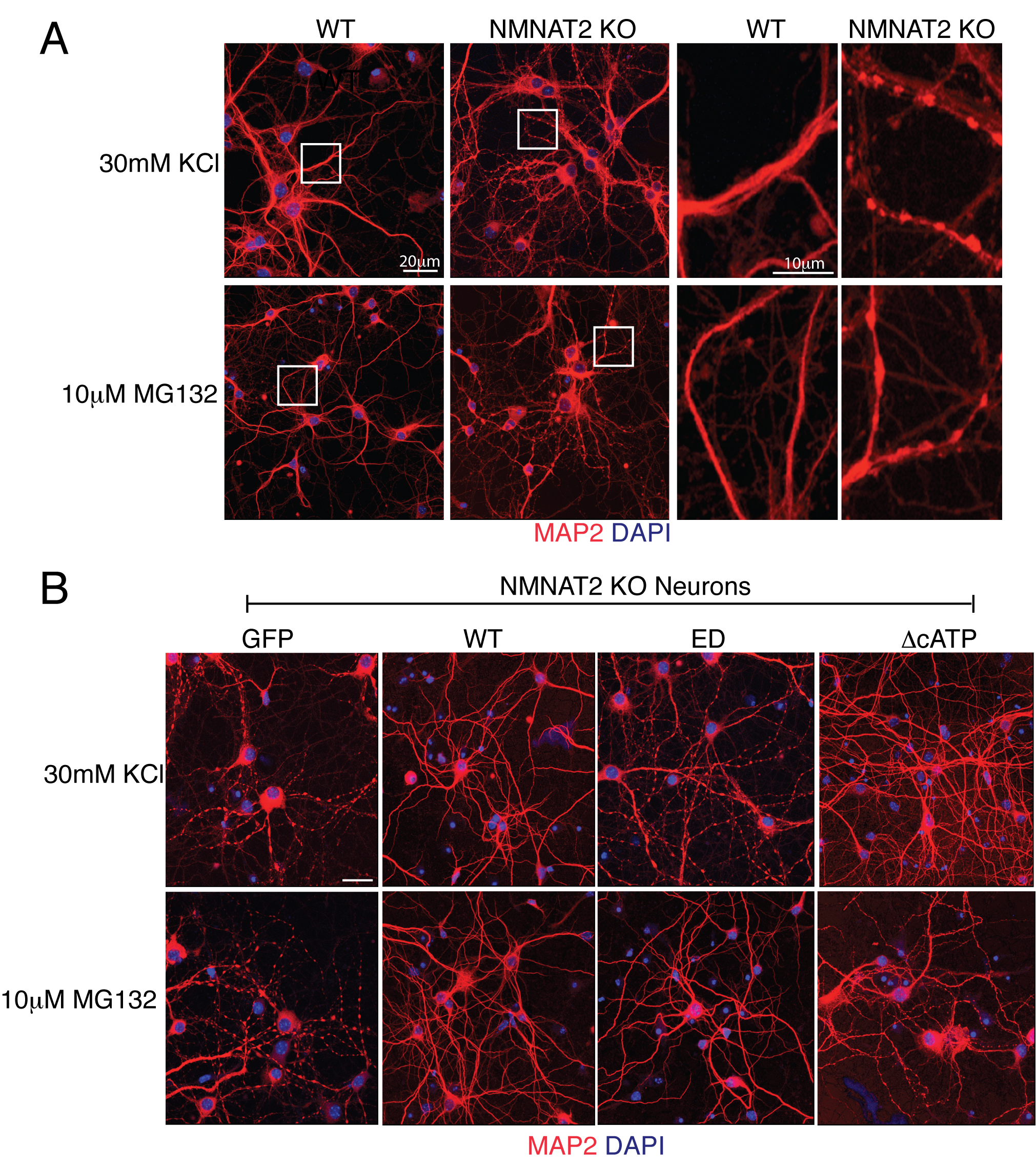

Supplement: S11 Fig — (A) Example images show that immunostaining with dendritic marker MAP2 (red) allowed us to visualize dying neurons with blebbing dendrites [113,114]. DAPI (blue)-stained nuclei. WT or NMNAT2 KO DIV14 neurons were treated with 30 mM KCl or 10 uM MG132 for 24 h. (B) Sample images of MAP2 immunostaining for NMNAT2 KO neurons overexpressing GFP or NMNAT2 wildtype or NMNAT2 variants. These neurons were treated with either KCl or MG132 as indicated. Raw data for S11A and S11B Fig can be found in S1 Data. (TIF) [file pbio.1002472.s012.tif]

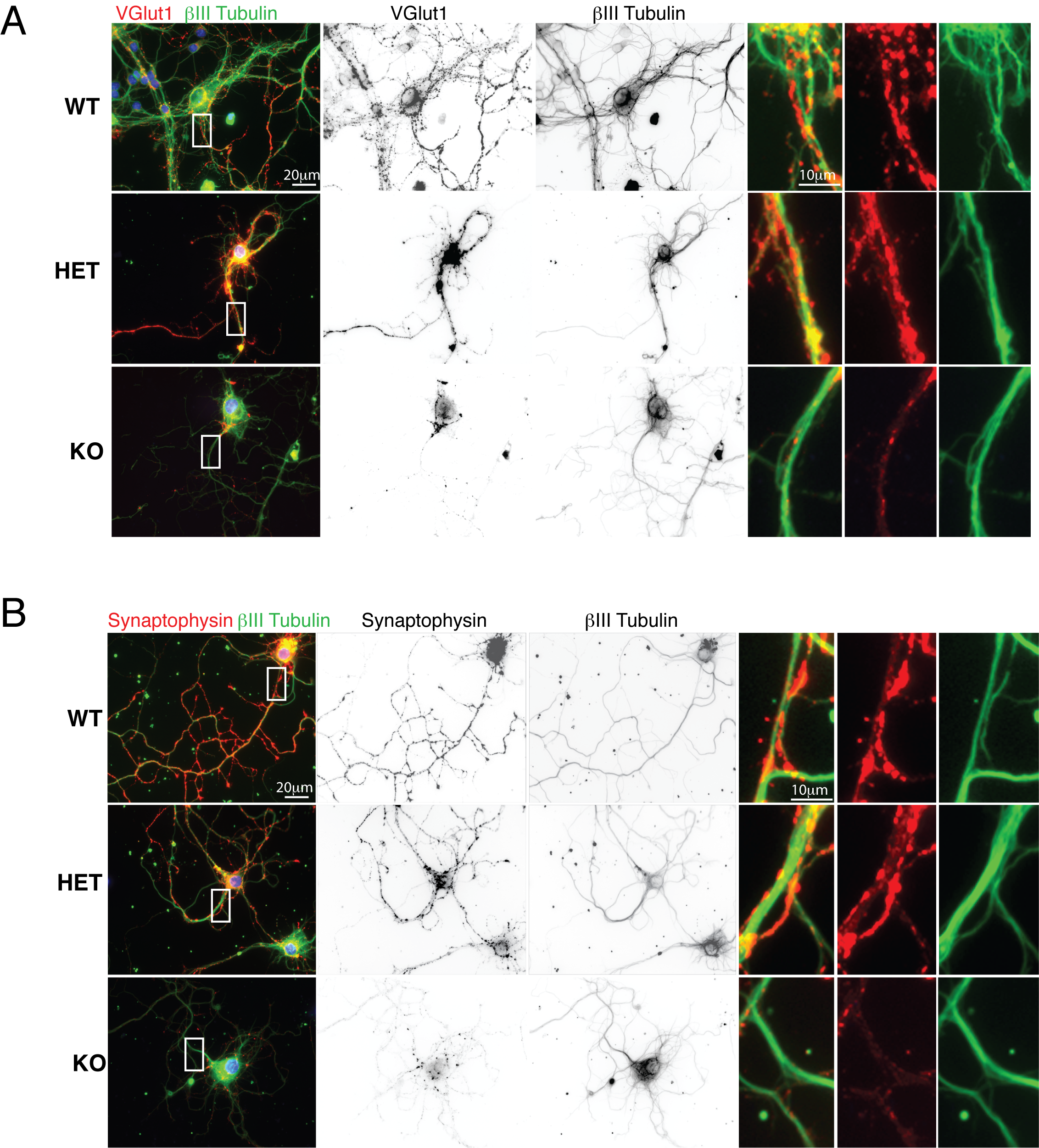

Supplement: S12 Fig — Example images of immunostaining of cultured NMNAT2 WT, HET and KO neurons at DIV10 with an axonal marker (BIII-Tubulin) and synaptic vesicle markers VGlut1 (A) or Synaptophysin (B). White box highlights region magnified in the right panels to show synaptic vesicle trafficking along axonal segments. Experiments were repeated three times. (TIF) [file pbio.1002472.s013.tif]

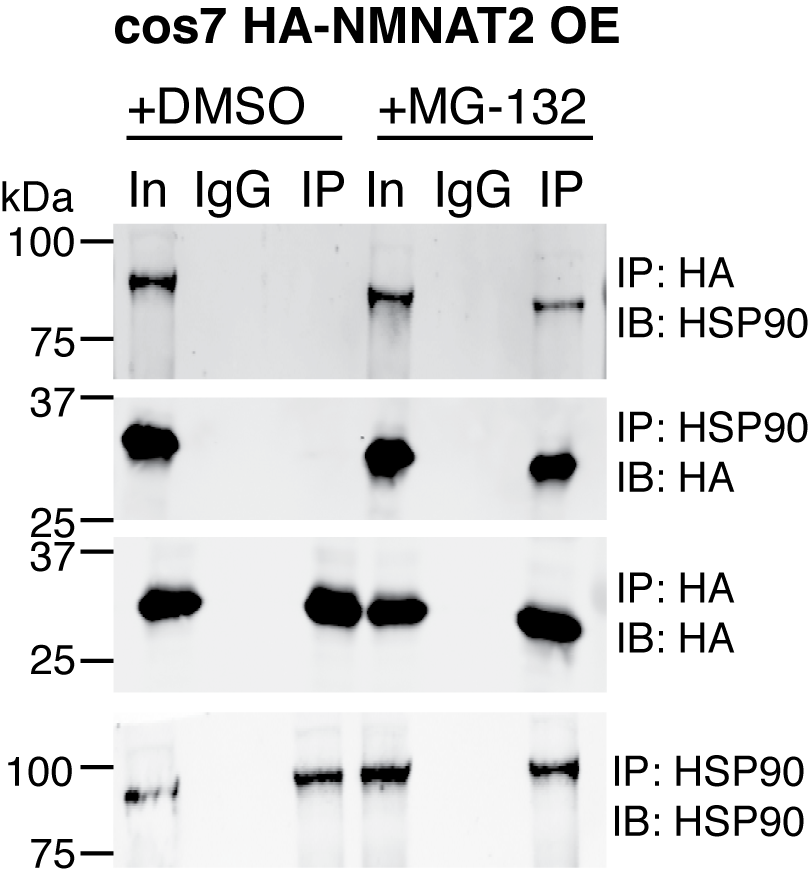

Supplement: S13 Fig — Cos7 cells were transfected with HA-NMNAT2, and after 48 h post transfection, they were treated with either DMSO or MG132 for 12 h. Lysates were used to immunoprecipitate with HSP90 or HA-NMNAT2 antibodies. IgG was used as negative control. In: Input; IP: Immunoprecipitate. (TIF) [file pbio.1002472.s014.tif]

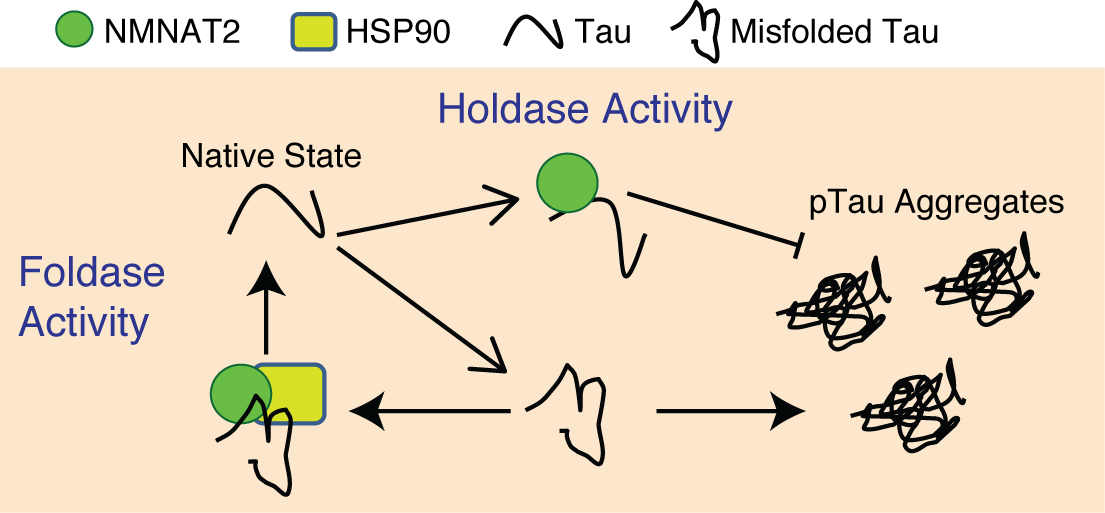

Supplement: S14 Fig — A simplified model of NMNAT2’s chaperoning activity. Upon proteotoxic stress, NMNAT2 binds to aggregation-prone proteins and maintains them in a nascent form while subsequent interactions with HSP90 promote refolding of aggregated substrates. (TIF) [file pbio.1002472.s015.tif]
