# Supplementary material for: NMNAT2:HSP90 Complex Mediates Proteostasis in Proteinopathies
Source: PLoS Biol. 2016 Jun 2;14(6):e1002472. doi: 10.1371/journal.pbio.1002472 (PMC4890852; doi:10.1371/journal.pbio.1002472)
Supplement: S1 Table — (DOCX) [file pbio.1002472.s016.docx]

| **Measure** | **Mean ± SD or n (%)** |
| --- | --- |
| **Demographics** |  |
| **Number of cases** | 541 |
| **Age of death, y** | 88.4 ± 6.7 |
| **Education, y** | 16.4 ± 3.6 |
| **Male** | 200 (37.0%) |
| **Cognitive Measures** |  |
| **Normal** | 172 (31.8%) |
| **MCI** | 139 (25.7%) |
| **AD** | 218 (40.3) |
| **Global Cognition** | -0.86 ± 1.14 |
| **Post-mortem indices** |  |
| **Global AD pathology** | 0.67 ± 0.60 |
| **PMI, h** | 7.2 ± 0.97 |
| **Transcripts** |  |
| **RIN** | 7.18 ± 0.97 |
| **NMNAT1** | 4.06 ± 1.75 |
| **NMNAT2** | 17.58 ± 8.83 |
