# Supplementary material for: NMNAT2:HSP90 Complex Mediates Proteostasis in Proteinopathies
Source: PLoS Biol. 2016 Jun 2;14(6):e1002472. doi: 10.1371/journal.pbio.1002472 (PMC4890852; doi:10.1371/journal.pbio.1002472)
Supplement: S2 Table — Linear regression models examining transcript levels in relation to the global cognition or global AD pathology measures. All analyses were adjusted for age of death, sex, postmortem interval, and RIN score. In a secondary analysis (*), the regression model was additionally adjusted for time from last clinical evaluation to death. (DOCX) [file pbio.1002472.s017.docx]

|  | **GLOBAL COGNITION** | | | **GLOBAL AD PATH** | | |
| --- | --- | --- | --- | --- | --- | --- |
|  | **Estimate** | **SE** | **P** | **Estimate** | **SE** | **P** |
| ***nmnat1*** | 0.02 | 0.03 | 0.37 | -0.005 | 0.01 | 0.58 |
| ***nmnat2*** | 0.03 | 0.007 | 0.00004 | -0.008 | 0.003 | 0.004 |
| ***nmnat2**** | 0.03 | 0.007 | 0.00006 | - | - | - |
