# Supplementary material for: NMNAT2:HSP90 Complex Mediates Proteostasis in Proteinopathies
Source: PLoS Biol. 2016 Jun 2;14(6):e1002472. doi: 10.1371/journal.pbio.1002472 (PMC4890852; doi:10.1371/journal.pbio.1002472)
Supplement: S3 Table — Braak: 6-many tangles, 0-no tangles; Plaque: 1-high plaque load, 4-no plaque. (DOCX) [file pbio.1002472.s018.docx]

| **Diagnosis Age PMI Braak Plaque** |
| --- |
| Control 83.1 2 1 1  Control >89 6 1 4  Control 86 8 2 4  Control 82.7 13 1 4  Control 77.4 12 0 4  Control 73 4 0 4  Control >89 12 2 3  AD 81 5.5 6 2  AD >89 18 6 2   AD 75 22 6 1  AD 68 11.5 6 1   AD 79 6 6 1   AD >89 6.25 6 2   AD 67 20.5 6 1   AD >89 5 6 2   NDAN >89 5 5 1  NDAN >89 8 6 1   NDAN 87.8 3 4 1  NDAN 87.6 10.5 5 2  NDAN 89 2.5 5 2   NDAN 91 3 days 5 1   NDAN >89 18 6 4 |
