# Supplementary material for: NMNAT2:HSP90 Complex Mediates Proteostasis in Proteinopathies
Source: PLoS Biol. 2016 Jun 2;14(6):e1002472. doi: 10.1371/journal.pbio.1002472 (PMC4890852; doi:10.1371/journal.pbio.1002472)
Supplement: S4 Table — % coverage gives the number of amino acids in sequenced peptides/the total number of amino acids in the protein. Accession gives the accession number from the NCBI nonredundant database with the organism set to Drosophila. *Name is the current accepted protein name as listed in Flybase.org. (DOCX) [file pbio.1002472.s019.docx]

| **% Coverage** | **Accession** | **Name** |
| --- | --- | --- |
| 16.16 | gi\|45551972 | nicotinamide mononucleotide adenylyltransferase |
| 24.836 | gi\|17647613 | multiprotein bridging factor 1, isoform A |
| 13.80 | gi\|17946488 | CG7903 |
| 3.20 | gi\|68051683 | CG1233 |
| 6.68 | gi\|21483492 | CG10764 |
| 2.62 | gi\|33636581 | CG32206 |
| 6.05 | gi\|4105248 | HCG-1 protein |
| 4.89 | gi\|17933552 | lava lamp |
| 4.38 | gi\|320542228 | paralytic, isoform BD |
| 8.95 | gi\|45549344 | PIP82 |
| 12.58 | RRRRRgi\|1335892 | ASH1 |
| 8.85 | gi\|17647335 | Gemin3 |
| 7.57 | gi\|19921616 | CG9246 |
| 11.45 | RRRRRgi\|24649281 | CG17083 |
| 5.54 | gi\|24585613 | clumsy, isoform A |
| 5.11 | gi\|281364798 | fatty acid (long chain) transport protein, isoform C |
| 8.65 | gi\|24652825 | megator |
| 3.91 | gi\|62484375 | CG2225, isoform B |
| 3.86 | RRRRRgi\|386769727 | tweek, isoform I |
| 5.76 | gi\|386765448 | Fmr1, isoform G |
| 2.33 | gi\|221513440 | Wnk, isoform A |
| 5.53 | gi\|38505146 | cytochrome P450 |
| 9.92 | gi\|320543067 | CG16718, isoform D |
| 6.87 | RRRRRgi\|386767818 | Sin3A, isoform G |
| 2.74 | RRRRRgi\|17352473 | fat |
| 9.21 | gi\|17647529 | **heat shock protein 83 (dHSP90 homolog)** |
| 5.42 | gi\|320543949 | O/E-associated zinc finger protein, isoform C |
