# Supplementary material for: NMNAT2:HSP90 Complex Mediates Proteostasis in Proteinopathies
Source: PLoS Biol. 2016 Jun 2;14(6):e1002472. doi: 10.1371/journal.pbio.1002472 (PMC4890852; doi:10.1371/journal.pbio.1002472)
Supplement: S5 Table — (DOCX) [file pbio.1002472.s020.docx]

| **Disease** | **Sample source** | **Cell type** | **Control** | **FC** | **P-value** | **Ref.** |
| --- | --- | --- | --- | --- | --- | --- |
| **Alzheimer’s Disease** | AD Patient | Pyramidal cell of Hippocampus | Aged Normal Control | -3.81 | 0.00003 | [1] |
|  | AD Patient | Pyramidal cell of Middle Temporal Gyrus | Aged Normal Control | -9.25 | 0.00006 | [2] |
| **FTLDU** | FTLDU Patient with Progranulin Mutation | Hippocampus | Normal Control | -6.22 | 0.003 | [3] |
| **Parkinson’s Disease** | PD patient | Medial Substantia Nigra | Control | -2.36 | 0.001 | [4] |
|  | PD patient | Lateral Substantia Nigra | Control | -2.53 | 0.004 | [5] |
| **Huntington’s Disease** | Grade4 HD patient | Caudate Nucleus | Control | -3.08 | 0.003 | [6] |
|  | Grade2 HD patient | Motor Cortex BA4 | Control | -1.36 | 0.018 | [6] |

References for S5 Table:

1. Liang WS, Dunckley T, Beach TG, Grover A, Mastroeni D, Walker DG, et al. Gene expression profiles in anatomically and functionally distinct regions of the normal aged human brain. Physiol Genomics. 2007;28(3):311-22.

2. Liang WS, Reiman EM, Valla J, Dunckley T, Beach TG, Grover A, et al. Alzheimer's disease is associated with reduced expression of energy metabolism genes in posterior cingulate neurons. Proc Natl Acad Sci U S A. 2008;105(11):4441-6.

3. Van Deerlin VM, Sleiman PM, Martinez-Lage M, Chen-Plotkin A, Wang LS, Graff-Radford NR, et al. Common variants at 7p21 are associated with frontotemporal lobar degeneration with TDP-43 inclusions. Nat Genet. 2010;42(3):234-9.

4. Lesnick TG, Papapetropoulos S, Mash DC, Ffrench-Mullen J, Shehadeh L, de Andrade M, et al. A genomic pathway approach to a complex disease: axon guidance and Parkinson disease. PLoS Genet. 2007;3(6):e98.

5. Moran LB, Duke DC, Deprez M, Dexter DT, Pearce RK, Graeber MB. Whole genome expression profiling of the medial and lateral substantia nigra in Parkinson's disease. Neurogenetics. 2006;7(1):1-11.

6. Hodges A, Strand AD, Aragaki AK, Kuhn A, Sengstag T, Hughes G, et al. Regional and cellular gene expression changes in human Huntington's disease brain. Hum Mol Genet. 2006;15(6):965-77.
